# Radiolabeled COX-2 Inhibitors for Non-Invasive Visualization of COX-2 Expression and Activity — A Critical Update

**DOI:** 10.3390/molecules18066311

**Published:** 2013-05-29

**Authors:** Markus Laube, Torsten Kniess, Jens Pietzsch

**Affiliations:** 1 Department Radiopharmaceutical and Chemical Biology, Institute of Radiopharmaceutical Cancer Research, Helmholtz-Zentrum Dresden-Rossendorf, Bautzner Landstrasse 400, 01328 Dresden, Germany; E-Mails: t.kniess@hzdr.de (T.K.); j.pietzsch@hzdr.de (J.P.); 2 Department of Chemistry and Food Chemistry, Technische Universität Dresden, 01062 Dresden, Germany

**Keywords:** cyclooxygenase, inhibitor, imaging, visualization, radionuclide, fluorine-18, carbon-11, radioiodine, COXIB, NSAID

## Abstract

Cyclooxygenase-2 (COX-2) is a key player in inflammation. Its overexpression is directly associated with various inflammatory diseases and, additionally, with several processes of carcinogenesis. The development of new selective COX-2 inhibitors (COXIBs) for use in cancer treatment is in the focus of the medicinal chemistry research field. For this purpose, a set of methods is available to determine COX-2 expression and activity *in vitro* and *ex vivo* but it is still a problem to functionally characterize COX-2 *in vivo.* This review focusses on imaging agents targeting COX-2 which have been developed for positron emission tomography (PET) and single photon emission computed tomography (SPECT) since 2005. The literature reveals that different radiochemical methods are available to synthesize COXIBs radiolabeled with fluorine-18, carbon-11, and isotopes of radioiodine. Unfortunately, most of the compounds tested did not show sufficient stability *in vivo* due to de[^18^F]fluorination or de[^11^C]methylation or they failed to bind specifically in the target region. So, suitable stability *in vivo*, matching lipophilicity for the target compartment and both high affinity and selectivity for COX-2 were identified as prominent criteria for radiotracer development. Up to now, it is not clear what approach and which model is the most suited to evaluate COX-2 targeting imaging agents *in vivo*. However, for proof of principle it has been shown that some radiolabeled compounds can bind specifically in COX-2 overexpressing tissue which gives hope for future work in this field.

## 1. Introduction

Since the enzyme cyclooxygenase-2 (COX-2) was discovered in the early 1990s, it has been intensively investigated and is still a challenging target for scientists from biology, pharmacy and medicine [1,2]. The reason for this is its prominent pathophysiological role in inflammation, inflammatory diseases, neurodegenerative disorders, and cancer [3] which all are closely associated with COX-2 overexpression. 

COX-2 is the inducible isoform of the cyclooxygenases (COX, EC 1.14.99.1) that catalyze the conversion of arachidonic acid into prostaglandin H_2_ (PgH_2_) [1,2,4,5,6]. This two-step reaction involves the cyclooxygenase/bis-oxygenation reaction at the cyclooxygenase-active site to form prostaglandin G_2_ (PgG_2_) and the subsequent reduction of the hydroperoxide at the peroxidase-active site to form prostaglandin H_2_ (PgH_2_) [7]. The subsequent conversion of PgH_2_ by tissue specific isomerases leads to prostaglandins and thromboxanes which act as autocrine and paracrine tissue hormones mediating a variety of physiological and pathophysiological processes. Both isoforms of COX, COX-1 and COX-2, share the same substrate, arachidonic acid, and show similar enzymatic activity but they differ significantly in their expression pattern and role in biological processes. COX-1 is constitutively expressed in most tissues and COX-1 derived prostaglandins control homeostatic functions, including gastric cytoprotection and hemostasis. In contrast, COX-2 is nearly absent in most normal tissues except brain, kidney, stomach, pancreas, uterus, ovary, and macrophages [8]. However, COX-2 is inducible and is highly expressed after an inflammatory or carcinogenic stimulation [3,6,7]. The overexpression of COX-2 leads to high levels of eicosanoids which mediate acute inflammation, pain and fever. Due to the fact that the COX-mediated reaction is the rate-limiting step of this process, COX-2 is called a key-player of inflammation. On the other hand, COX-2 overexpression is also associated with chronic inflammatory diseases, e.g., rheumatoid arthritis and ulcerative colitis, and neurodegenerative diseases like Parkinson’s or Alzheimer’s disease. In this regard, COX-2 is discussed as an important target for the prevention and treatment in this field [3,9]. Beside COX-1 and COX-2, a splice variant of COX-1, COX-1b, was reported which does not result in translation of a functionally active enzyme in humans [10,11].

The inhibition of the cyclooxygenase-2 reaction results in anti-inflammatory, analgesic and anti-pyretic effects and can be achieved regarding the concomitant inhibition of COX-1 unselectively, by traditional nonsteroidal anti-inflammatory drugs (NSAIDs), e.g., aspirin, ibuprofen, or selectively, by selective COX-2 inhibitors (COXIBs), e.g., celecoxib, valdecoxib or rofecoxib [12,13,14]. With the exception of aspirin, selective and unselective COX inhibitors bind competitively in the cyclooxygenase active site and hinder in this way the conversion of arachidonic acid. COX-2-selectivity is specified by the inhibitor’s structure and the resulting possibility to interact with a side pocket of COX-2 which is not accessible in COX-1. Unfortunately, both ways to inhibit cyclooxygenase activity in a long-term matter are accompanied by adverse side effects: unselective COX-inhibition and thereby inhibition of COX-1 results, e.g., in gastrointestinal toxicity like ulceration and inhibition of COX-2—selective or unselective—results in an elevated cardiovascular risk. Today, most selective COX-2 inhibitors are withdrawn from the market or used with caution [15]. However, there is still an ongoing research for new and safer COX-2 selective inhibitors [16] due to the huge market for analgesic drugs as well as their potential use in diagnosis and treatment of the above mentioned diseases. Since COX-2 overexpression was also shown in cancer and is assumed to be implicated in inflammogenesis of cancer [11,17,18,19,20,21,22,23,24], it came more and more in the focus as a diagnostic marker and therapeutic target in oncology. Additionally, COX-2 was identified as predictive marker in certain tumor entities [25,26].

The accurate determination of COX-2 expression *in vivo* is one crucial requirement to detect COX-2 related disorders and for the development of COX-2 targeted therapies. Although nowadays a variety of methods are known for *in vitro* assessment of COX-2 expression and activity, the functional characterization of the enzyme *in vivo* is still an unsolved problem. Today, the latter is commonly done by *ex vivo* laboratory analysis of patients specimen invasively obtained from biopsies. A non-invasive molecular imaging method would overcome this problem [27,28,29,30,31]. In this regard, the radionuclide-based imaging of COX-2 expression and activity has been discussed to be advantageous for several reasons. By using radiolabeled COX-2 inhibitors with both, high affinity and selectivity, it would be possible to obtain non-invasive and repeatable *in vivo* data of the functional COX-2 expression level in manifold diseases over the time that means during their manifestation, progression, and under targeted therapy. This is much more important since the involvement of COX-2 in a number of diseases is still not fully understood and, hence, non-invasive imaging would help to get a more detailed insight into the involvement of COX-2 in these pathophysiological situations [27,28,29,31,32,33,34,35,36,37,38,39]. The pharmacological data achievable from the radiotracers could help to understand the physiological behavior and metabolic pathways of the compounds itself [30,40,41]. The measurement of the occupancy of COX-2 binding sites would allow their use as surrogate marker for the *in vivo* evaluation of novel COX-2 inhibitors and their use in dose-escalation studies [35]. Furthermore, radionuclide-based non-invasive imaging of COX-2 is of great interest for the clinical diagnosis due to the possibility to quantify the COX-2 expression since its expression level in diseases is discussed as biological marker for the early diagnosis, the monitoring of disease progression, and the evaluation of therapeutic efforts [38,42,43,44,45,46]. This includes also the monitoring of inflamed processes [47], as well as the detection of chronically inflamed tissue [48] and, on the other hand, early-staged or advanced tumors [48,49,50]. In this regard, the visualization of COX-2 expression would be valuable for the predictive evaluation of COX-targeted therapies [51] as well as for the determination of COX-2 expression in individual patients to decide whether COX-2 is a suitable molecular target for an individualized treatment [9]. So, the physician would furthermore have prior to the therapy a tool to weigh up the possible benefit of the treatment with COXIBs against the elevated cardiovascular risk associated with these drugs [13,52,53]. Finally, non-invasive imaging of COX-2 expression would also provide useful information to validate the COX-2 response with anti-COX-2-based clinical studies [54], and to relate the COX-2 inhibition with therapeutic effects [47]. 

Different strategies have been followed to determine the COX-2 expression *in vivo* with radiotracers indirectly by visualization of COX-2 related effects or directly by using radiolabeled substrates and inhibitors. Exemplarily, the visualization of effects associated with COX-expression is described by Shimizu *et al.* for non-small cell lung carcinoma patients [55]. As one result, a strong correlation was demonstrated between the maximum standardized uptake value (SUV_max_) of [^18^F]fluorodeoxyglucose ([^18^F]FDG)-PET scans and the expression of COX-2 which was examined afterwards by immunohistochemical tissue staining of resected human lung adenocarcinomas. This example shows that by means of a routine [^18^F]FDG-PET evidence could be given for the occurrence of further disease related biomarkers, e.g., COX-2 expression. In this way, this information about the malignancy in special cases may assist the further treatment planning.

Concerning the literature about radiolabeled COX-2 inhibitors up to 2004 the reader is referred to the excellent review by de Vries. [3]. In brief, the natural substrate of cyclooxygenase-2, arachidonic acid, itself was labeled with carbon-11 by Channing *et al.* in 1993 but this strategy turned out to be not suitable for positron emission tomography (PET) imaging due to incorporation of the tracer into phospholipids in the brain [56,57]. The first attempt to radiolabel a COX inhibitor was performed by del Rosario in 1996 who synthesized [^11^C]indomethacin methyl ester as reported in a short abstract not showing *in vivo* evaluation data [58]. The first report about *in vitro* and *in vivo* evaluation of a COX-2 inhibitor by PET followed five years later, in 2002, with the synthesis of [^18^F]SC58125 by McCarthy *et al.* [32]. Then, 2003 the synthesis of another COX-2 selective inhibitor, [^18^F]desbromo-DuP-697, for PET was reported by de Vries *et al.* [27]. Attempts to synthesize radiotracers for single photon emission computed tomography (SPECT) began in 2002 with the synthesis of the nonselective COX-inhibitor Tc-99m-diflunisal [59] and were followed by the synthesis of ^99mT^c-labeled celecoxib in 2004 [54]. Although some radiolabeled COX-inhibitors were described at that time, all these imaging agents failed to show specific binding *in vivo*. 

## 2. Following Developments

From 2005, more and more radiolabeled COX-2 selective inhibitors emerged in the literature. These radiotracers were in primary radiolabeled with positron emitting nuclides for imaging with PET but there also exist reports about radiolabeled compounds with isotopes of iodine suitable for SPECT imaging. Therefore, this review focusses on imaging agents targeting COX-2 which have been developed for PET and SPECT since 2005.

### 2.1. COX-2 Inhibitors Radiolabeled with PET Nuclides

#### 2005

**Scheme 1:**
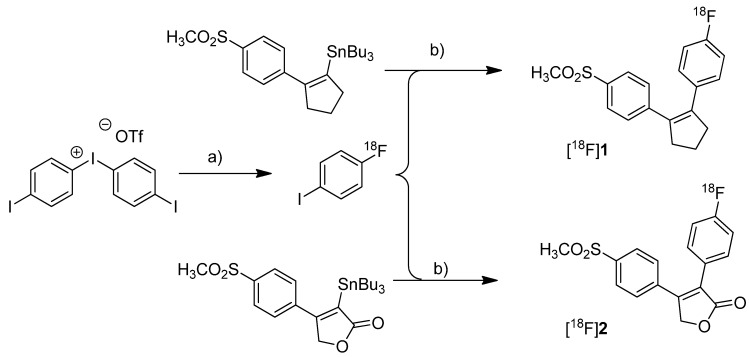
Synthesis of [^18^F]**1** and [^18^F]**2**.

In 2005, the synthesis of three fluorine-18- and seven carbon-11-labeled COX-2 inhibitors as well as one carbon-11-labeled COX-1 inhibitor was reported. Three of these compounds were investigated *in vivo*. Wuest *et al.* presented the radiosynthesis of two COX-2 inhibitors, 1-[^18^F]fluoro-4-(2-(4-methylsulfonyl)phenyl)cyclopent-1-enyl)benzene ([^18^F]**1**, Scheme 1) and 3-(4-[^18^F]fluorophenyl)-4-(4-(methylsulfonyl)phenyl)-5*H*-furan-2-one ([^18^F]**2**, Scheme 1), possessing a cyclopentene and a 2-(5*H*)furanone ring as carbocyclic core [41]. Compound **2** is a fluoro derivative of rofecoxib.

*Reagents and conditions*: (**a**) [^18^F]KF, K_222_, K_2_CO_3_, DMF, 120 °C; (**b**) different conditions, optimized: Pd_2_(dba)_3_/P(o-tolyl)_3_/CuI, DMF-toluene, 65 °C, 20 min.

**Synthesis:** The radiolabeling procedure was performed *via* Stille reaction. In detail, the tributyl stannyl precursor was reacted with 4-[^18^F]fluoroiodobenzene which was obtained from an iodonium salt by reaction with [^18^F]fluoride. Using Pd_2_(dba)_3_/P(*o*-tolyl)_3_/CuI as catalyst system in DMF/toluene at 65 °C for 20 min, the Stille cross-coupling gave compound [^18^F]**1** and [^18^F]**2** in an optimized radiochemical yield of 68% and 93% as determined by HPLC, respectively. ***In vitro*/*In vivo*:** Further information about *in vitro* or *in vivo* evaluation of the radiotracers was not given. 

**Scheme 2:**
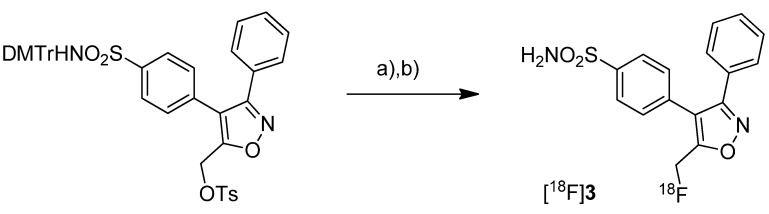
Synthesis of [^18^F]**3**.

Toyokuni *et al.* presented the synthesis of 4-(5-[^18^F]fluoromethyl-3-phenylisoxazol-4-yl)-benzenesulfonamide ([^18^F]**3**, Scheme 2), a [^18^F]fluorinated analog of valdecoxib, and the results of a preliminary evaluation by PET [33]. 

*Reagents and conditions*: (a) [^18^F]KF,K_222_, K_2_CO_3_, MeCN, 90 °C, 5 min; (b) 40% TCA in MeCN, 90 °C, 5 min.

**Synthesis:** The radiosynthesis started from a 4,4'-dimethoxytrityl-sulfonamide protected *O*-tosylated precursor and was performed by [^18^F]fluoride-for-tosylate exchange followed by acidic deprotection and purification by preparative HPLC. This gave chemically and radiochemically pure compound [^18^F]**3** in 40% decay corrected radiochemical yield within 120 min after end of bombardment with a specific activity of about 74 GBq/µmol. *In vitro*: *In vitro* experiments for this tracer were not reported. *In vivo*: Instead, a preliminary small animal PET study using a normal mouse as well as a PET study using a vervet monkey was performed. The monkey whole-body biodistribution data allowed a dosimetry calculation for a 70 kg adult and an injection of 370 MBq [^18^F]**3**. This revealed that the maximum absorbed dose found in the urinary bladder would be below the recommended dose limitation ratio. Finally, further detailed evaluation of the radiotracer was hampered due to substantial de[^18^F]fluorination which was observed in mouse as well as in monkey by fluorine-18-activity enrichment in the skeleton after 15 min and 45 min, respectively. 

**Scheme 3:**
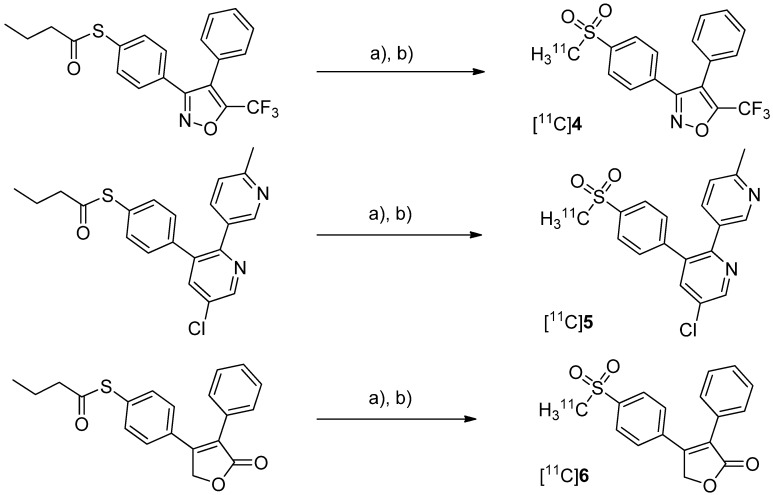
Synthesis of [^11^C]**4**–**6**.

**Scheme 4:**
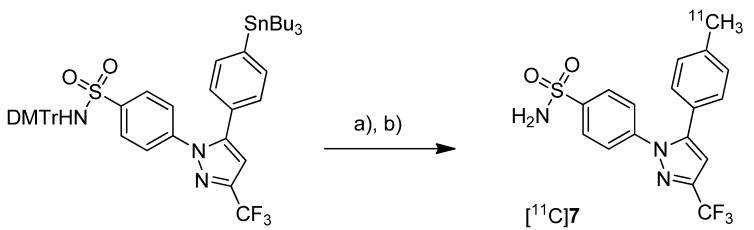
Synthesis of [^11^C]**7**.

After having reported the synthesis of 3-(4-([^11^C]methylsulfonyl)phenyl)-4-phenyl-5-(trifluoromethyl)isoxazole ([^11^C]**4**, Scheme 3) [47] and the synthesis of the celecoxib analog 4-(5-(4-[^11^C]methylphenyl)-3-(trifluoromethyl)-1*H*-pyrazol-1-yl)benzenesulfonamide ([^11^C]**7**, Scheme 4) [60] in conference contributions before, a detailed description of both radiotracers followed in 2005. 

*Reagents and conditions*: (**a**) [^11^C]CH_3_I, TBAOH for [^11^C]**4** and [^11^C]**5** pyrrolidine for [^11^C]**6**, THF, r.t., 5 min; (**b**) Oxone^®^, MeOH/H_2_O (1:1), 70 °C and 2 min for [^11^C]**4** and [^11^C]**6**, 75 °C and 3 min for [^11^C]**5**.

Majo *et al.* presented the synthesis of three carbon-11-labeled COX-2 inhibitors thus exemplifying a new strategy for the synthesis of aryl [^11^C]methylsulfones [42]. 5-chloro-6'-methyl-3-(4-([^11^C]methylsulfonyl)phenyl)-2,3'-bipyridine ([^11^C]**5**, Scheme 3) and 4-(4-([^11^C]methylsulfonyl)-phenyl)-3-phenylfuran-2(5*H*)-one ([^11^C]**6**, Scheme 3) are the isotopically labeled analogs of eterocoxib and rofecoxib. **Synthesis:** The synthesis of all three compounds started with a thiobutyrate ester precursor which was converted into the radiolabeled COX-2 inhibitor by unraveling the free thiol under basic conditions followed by *S*-[^11^C]methylation and final oxidation of the thiomethyl ether to the methylsulfonyl moiety. In case of compound [^11^C]**4 **and [^11^C]**5**, tetrabutylammonium hydroxide in THF was used for the cleavage of the thiobutyrate ester. This failed for the synthesis of [^11^C]**6** due to concomitant cleavage of the γ-butyrolactone ring. Therefore, in case of compound [^11^C]**6** an excess of pyrrolidine in DMF was successfully used instead. This strategy provided compounds [^11^C]**4**, [^11^C]**5** and [^11^C]**6** after semi-preparative HPLC in a total synthesis time of 30 min and a decay corrected radiochemical yield of 37%, 28%, and 20% (based on [^11^C]methyl iodide), respectively. The specific activity of [^11^C]**4** was determined to be 74 ± 9.25 GBq/µmol. ***In vitro*/*In vivo*:** Unfortunately, no further *in vitro* or *in vivo* evaluations have been presented in this work. [^11^C]**6** was synthesized and evaluated by de Vries *et al.* in 2008 [29]. 

Prabhakaran *et al.* reported the synthesis of 4-(5-(4-[^11^C]methylphenyl)-3-(trifluoromethyl)-1*H*-pyrazol-1-yl)benzenesulfonamide ([^11^C]**7**, Scheme 4), the isotopically labeled carbon-11 analog of celecoxib [61].

*Reagents and conditions*: (**a**) Pd_2_(dba)_3_, P(*o*-tolyl)_3_, [^11^C]CH_3_I, DMF, 135 °C, 4 min; (**b**) 20% TFA in DCM, 60 °C, 5 min.

**Synthesis:** The synthesis towards the [^11^C]methyl substituted 4-tolyl moiety of [^11^C]**7** started from a stannylated and DMT-sulfonamide protected precursor by *C*-[^11^C]methylation and final acidic deprotection. The initial labeling reaction was achieved by trapping [^11^C]methyl iodide in an argon purged Pd_2_(dba)_3_/P(*o*-tolyl)_3_ solution of DMF, addition of the precursor and subsequent reaction at 135 °C for 4 min. The following deprotection with 20% trifluoroacetic acid (TFA) in dichloromethane and RP-HPLC purification gave compound [^11^C]**7** in 8 ± 2% (n = 6) decay corrected radiochemical yield (based on [^11^C]methyl iodide) with a specific activity of 39.96 ± 6.66 GBq/µmol and a radiochemical purity >99%. For transmetallation of the tin precursor it was found to be essential to use reagents of high purity. The authors also pointed to the need to detect impurities of tin and palladium which were estimated to be less than 39 and 31 ppb using atomic absorption spectroscopy. ***In vitro*/*In vivo*:** Also in this work further *in vitro* or *in vivo* evaluation was not presented.

**Scheme 5:**
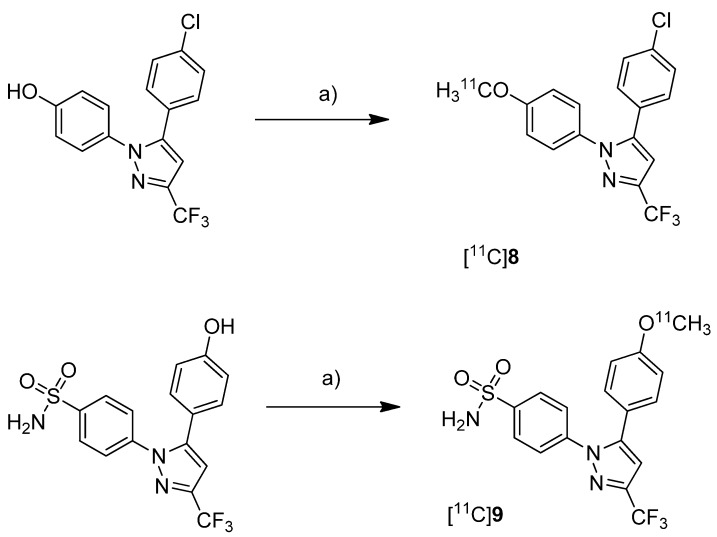
Synthesis of [^11^C]**8** and [^11^C]**9**.

Fujisaki *et al.* reported the radiosynthesis of 5-(4-chlorophenyl)-1-(4-[^11^C]methoxyphenyl)-3-(trifluoromethyl)-1*H*-pyrazole ([^11^C]**8**, Scheme 5), a COX-1 selective inhibitor (IC_50_(COX-1) = 9 nM; IC_50_(COX-2) = 6.3 µM [62]), and 4-(5-(4-[^11^C]methoxyphenyl)-3-(trifluoromethyl)-1H-pyrazol-1-yl)benzenesulfonamide ([^11^C]**9**, Scheme 5) a [^11^C]methoxy analog of celecoxib (IC_50_(COX-1) = 2.6 µM; IC_50_(COX-2) = 8 nM [62]) [51]. Both compounds were evaluated *in vivo* and *ex vivo.* **Synthesis**: The radiolabeling procedure was similar for both compounds and started each time from the phenolic hydroxy precursor. The tracers were synthesized by *O*-[^11^C]methylation and the reaction conditions were optimized using [^11^C]methyl iodide as well as [^11^C]methyl triflate. The best results were obtained by using [^11^C]methyl triflate and an equimolar amount of base with respect to the hydroxy precursor in acetone. This protocol yielded [^11^C]**8** and [^11^C]**9** in 74.5% and 46.6% decay corrected radiochemical yield (based on [^11^C]methyl triflate) with a specific activity of 71–125 GBq/µmol and 27–61 GBq/µmol, respectively, and radiochemical purity >99% after 20 min total synthesis time. The log *P*_7.4_ was determined to be 3.46 for [^11^C]**8** and 3.06 for [^11^C]**9**. *In vitro*: *In vitro* experiments for these tracers were not reported. *In vivo*/*Ex vivo*: For *in vivo* experiments male Donryu rats were used bearing subcutaneously an AH109A ascetic hepatoma cell xenograft tumor. COX-2 expression of the AH109A tumor cells was confirmed by Western blot analysis. *In vivo* experiments revealed for [^11^C]**8** and [^11^C]**9** a high extend of non-specific binding. The COX-2 selective inhibitor [^11^C]**9** showed high levels of radioactivity in the blood, a low uptake into the AH109A tumor and the brain and a much slower blood clearance in comparison to [^11^C]**8**. Metabolite analysis revealed that >93% of each compound remained unchanged in the brain and in the AH109A tissue after 30 min. The blocking studies by coinjection of [^11^C]**9** with 8 or indomethacin as well as carrier-loading did not lower the uptake in any tissues except spleen and blood in case of carrier loading. In contrast, in few tissues radioactivity levels even increased. Although *ex vivo* autoradiograms of rat brain showed regional differences in tracer distributions similar to the expression pattern of cyclooxygenases a selective blocking could not be shown. 

*Reagents and conditions*: (**a**) different conditions, optimized: [^11^C]CH_3_OTf, aq. NaOH, acetone, r.t., 2 min.

**Scheme 6:**
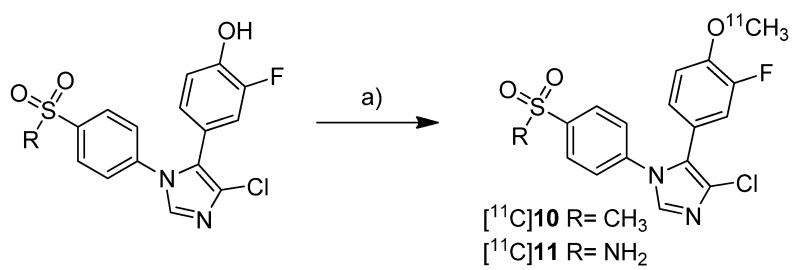
Synthesis of [^11^C]**10**–**11**.

Together with the synthesis of [^11^C]**8**–**9**, Kawamura *et al.* presented the synthesis of [^11^C]**10-11** (Scheme 6) as part of a conference contribution [63]. 4-Chloro-5-(3-fluoro-4-methoxyphenyl)-1-(4-(methylsulfonyl)phenyl)-1*H*-imidazole (**10**) and 4-(4-chloro-5-(3-fluoro-4-methoxyphenyl)-1*H*-imidazol-1-yl)benzenesulfonamide (**11**) were described as selective COX-2 inhibitors with IC_50_(COX-2) of 4 nM and IC_50_(COX-1) >10 µM for **10** and 5 nM and 3.3 µM for **11**, respectively [64].

*Reagents and conditions*: (**a**) different conditions, optimized: [^11^C]CH_3_OTf, aq. NaOH, acetone, r.t. or 120 °C, 1 min.

The reported reaction conditions [51] were used that gave [^11^C]**10** in 64.8–78.2% (n = 4) and [^11^C]**11** in 66.1–74.1% (n = 4) decay corrected radiochemical yield (based on [^11^C]methyl triflate).

#### 2006

In 2006, the synthesis of one fluorine-18-labeled and three carbon-11-labeled COX-2 inhibitors was reported whereby the latter were investigated *in vivo*. 

**Scheme 7:**
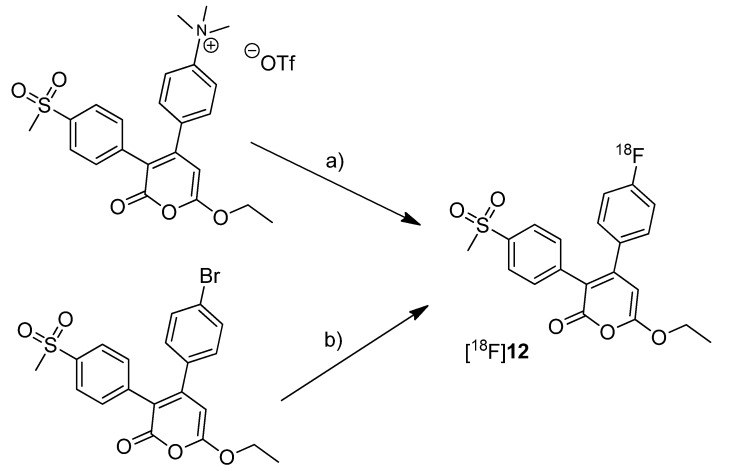
Synthesis of [^18^F]**12**.

Tian and Lee reported the synthesis of the fluorine-18-labeled COX-2 inhibitor 6-ethoxy-4-(4-[^18^F]fluorophenyl)-3-(4-(methylsulfonyl)phenyl)-2*H*-pyran-2-one ([^18^F]**12**, Scheme 7) (IC_50_(COX-2) = 0.1 µM; SI = IC_50_(COX-2)/IC_50_(COX-1) = 2880 [65]) [28].

*Reagents and conditions*: (**a**) [^18^F]KF,K_222_, K_2_CO_3_, MeCN, 130 °C, 20 min; (**b**) [^18^F]KF,K_222_, K_2_CO_3_, DMSO, 160 °C, 30 min.

**Synthesis:** The radiosynthesis was performed starting from the trimethylammonium phenyl precursor as well as the bromo phenyl precursor by nucleophilic substitution with [^18^F]fluoride. For the trimethylammonium precursor, the optimized labeling condition was determined to be 130 °C in acetonitrile followed by semi-preparative HPLC that yielded [^18^F]**12** in 14.6 ± 3.3% decay corrected yield with a specific activity of 18.02 ± 3.15 GBq/µmol after 60–70 min total synthesis time. Radiofluorination of the bromo phenyl precursor was performed at 160 °C in DMSO but this method gave the radiotracer only in 4% radiochemical yield and with low specific activity due to troublesome separation of the brominated precursor from the [^18^F]fluorinated product by semi-preparative HPLC. ***In vitro*/*In vivo*:** The *in vitro* or *in vivo* evaluation of [^18^F]**12** was not presented.

**Scheme 8:**
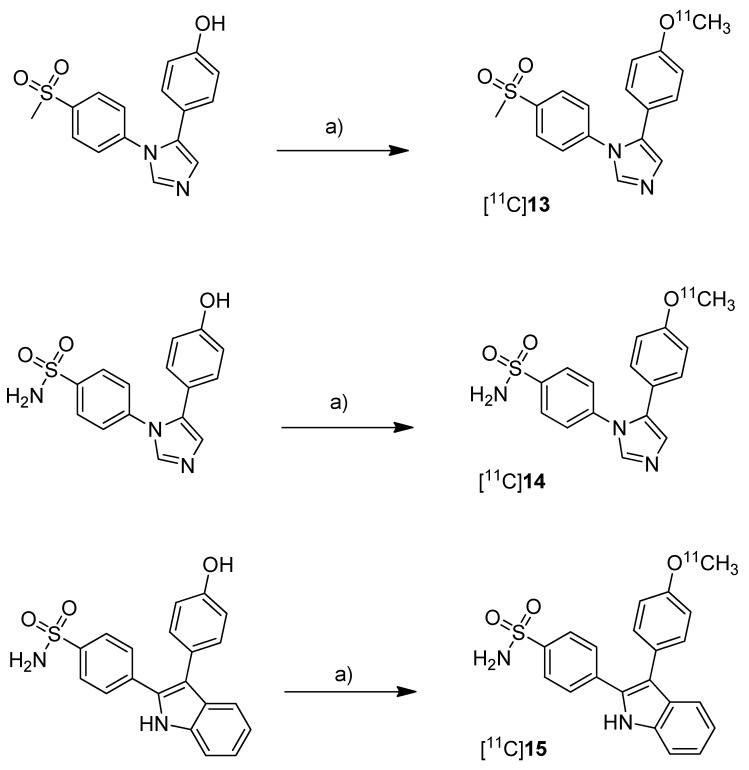
Synthesis of [^11^C]**13**–**15**.

One year after the synthesis of [^11^C]**9**, Tanaka and coworkers published their results regarding the synthesis of three COX-2 inhibitors labeled with carbon-11 and the *in vitro*, *in vivo* and *ex vivo* evaluation [43]. Non-radioactive analogs were previously reported to have high affinity and to be highly selective towards COX-2 with IC_50_(COX-2) and IC_50_(COX-1) of 4 nM and > 10 µM for 5-(4-methoxyphenyl)-1-(4-(methylsulfonyl)phenyl)-1*H*-imidazole (**13**, Scheme 8) [64], 5 nM and > 3.3 µM for 4-(5-(4-methoxyphenyl)-1*H*-imidazol-1-yl)benzenesulfonamide (**14**, Scheme 8) [64] and 0.006 nM and > 10 µM for 4-(3-(4-methoxyphenyl)-1*H*-indol-2-yl)benzenesulfonamide (**15**, Scheme 8) [66].

*Reagents and conditions*: (**a**) different conditions: optimized: [^11^C]CH_3_OTf, aq. NaOH, acetone, r.t., 1 min.

**Synthesis:** The radiolabeling procedure started for all three compounds from the phenolic hydroxy precursor by O-[^11^C]methylation. Using [^11^C]methyl triflate in acetone with an equimolar amount of NaOH as the base and subsequent purification with semi-preparative HPLC gave [^11^C]**13**, [^11^C]**14** and [^11^C]**15** in decay corrected radiochemical yields (based on [^11^C]methyl triflate) of 75.1% (n = 1),66.1–74.1% (n = 3) and 17.5–25.1% (n = 3), respectively, with a specific activity of 36–264 GBq/µmol within 20 min total preparation time. The log P_7.4_ values were found to be between 1.89 and 2.33. **In vivo/Ex vivo:** AH109A tumor bearing mice as described by Fujisaki et al. [51] were used for the *in vivo* experiments. The tissue distribution studies revealed that all three compounds showed the highest uptake in the liver. Some differences of the single radiotracers in blood uptake and organ distribution were found but there was no COX-specific binding observed in COX-2 expressing tissues such as brain, kidney, heart and lung, which was confirmed by blocking experiments. Also the tumor uptake was low showing no COX-specific blocking effects. Regional differences in brain uptake of [^11^C]**13** were not further investigated. In contrast to compound [^11^C]**13** and [^11^C]**14** which were detected more or less unchanged in plasma and AH109A tumor tissue with more than 90% after 30 min, [^11^C]**15** was found to be relatively unstable. 54% and 80% of original [^11^C]**15** was detected after 30 min in plasma and in the AH109A tumor, respectively. Interestingly, [^11^C]**14** and [^11^C]**15** showed only very low uptake in the brain although lipophilicity would allow crossing the blood-brain barrier as discussed by the authors. **In vitro:** For this, there were performed transcellular transport studies using human cells overexpressing the multidrug resistance protein 1 or permeability glycoprotein 1 (P-gp) and the P-gp inhibitor cyclosporine A (CsA). Although compound [^11^C]**15** was shown to be an **in vitro** substrate of P-gp which is responsible for the efflux of different drugs out of the brain, inhibition of P-gp **in vivo** did not alter the brain uptake, a behavior that still needs to be elucidated.

#### 2007

**Scheme 9:**
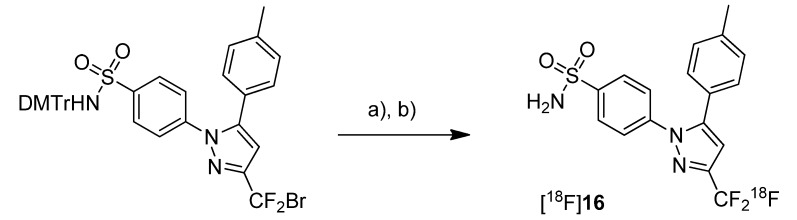
Synthesis of [^18^F]**16**.

In 2007, the synthesis of only one fluorine-18-labeled COX-2 inhibitor was presented by Prabhabkaran *et al.* [34,44]. The synthesized fluorine-18-labeled analog of celecoxib, 4-(5-(methylphenyl)-3-([^18^F]trifluoromethyl)-1*H*-pyrazol-1-yl)benzenesulfonamide ([^18^F]**16**, Scheme 9), was evaluated *in vivo* in mice and baboons.

*Reagents and conditions*: (**a**) [^18^F]TBAF, DMSO, 135 °C, 20 min; (**b**) 50% TFA in DCM, 60 °C, 5 min.

**Synthesis:** The radiosynthesis of [^18^F]**16** was performed by [^18^F]fluorine-for-bromine exchange starting from the bromodifluoro-methyl precursor. Because [^18^F]KF-Kryptofix was ineffective even with rigorous reaction conditions, tetra-*n*-butylammonium [^18^F]fluoride ([^18^F]TBAF) was used for labeling. Best yields were achieved at 135 °C in DMSO for 20 min and subsequent deprotection with TFA in dichloromethane. This method gave [^18^F]**16** after semi-preparative HPLC in an optimized radiochemical yield of 10 ± 2% (n = 6) with a chemical and radiochemical purity of > 99% but only low specific activity of 4.44 ± 1.48 GBq/µmol. [^18^F]**16** showed no de[^18^F]fluorination in pure ethanol but a low rate of de[^18^F]fluorination in 10% ethanol-saline solution with 93.5% of intact compound after 4 h. *In vivo*: PET studies in male Sprague-Dawley rats were performed with [^18^F]**16** that showed slight uptake in the brain and the heart but also a significant skeleton uptake due to de[^18^F]fluorination of the radiotracer within 60–120 min. Although this indicated that [^18^F]**16** is unsuitable for imaging COX-2 expression, a PET study in a baboon was performed due to the lower de[^18^F]fluorination rates in baboon compared to rodents. These PET studies revealed that [^18^F]**16** penetrated the blood brain barrier, accumulated in the brain and that the distribution of the tracer was consistent with the known distribution of COX-2. As expected, the de[^18^F]fluorination rate was lower in baboon as shown by a 3–4 times lower brain to skull uptake ratio compared to tissue and skeleton in rodents. Unfortunately, the radiotracer underwent fast metabolism so that already within 60 min 83% of [^18^F]**16** were degraded mainly to polar metabolites. 

#### 2008

**Scheme 10:**
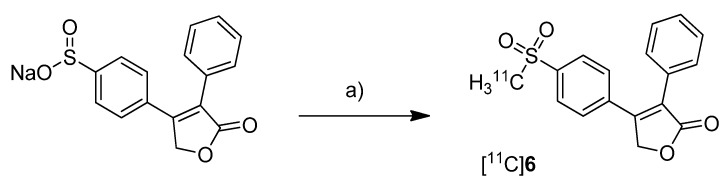
Synthesis of [^11^C]**6**.

In 2008, the synthesis and *in vivo* evaluation of further three carbon-11-labeled COX-2 inhibitors was described. Five years after the synthesis of [^18^F]DuP-697, de Vries *et al.* presented a new synthesis of [^11^C]**6** (Scheme 10) the isotopically labeled analog of rofecoxib, and its *in vivo* evaluation because this was not performed yet [29]. Because rofecoxib was shown to penetrate the brain resulting in high brain concentrations compared to other COXIBs, [^11^C]**6** was initially evaluated as a brain tracer for COX-2 expression.

*Reagents and conditions:* (**a**) [^11^C]CH_3_I, DMF, 90 °C, 4 min.

**Synthesis:** The radiosynthesis of [^11^C]**6** was performed starting from a different precursor than Majo *et al.* [42], the sulfinate, by reaction with [^11^C]methyl iodide in DMF at 90 °C for 4 min and subsequent semi-preparative HPLC purification. This yielded [^11^C]**6** in > 99% radiochemical purity within 40 min in 57 ± 9% (n = 23) decay corrected radiochemical yield (based on [^11^C]methyl iodide) and a specific activity of 14 ± 8 GBq/µmol. *In vivo*/*Ex vivo*: Male Wistar rats were used for the evaluation of [^11^C]**6**. The distribution in the brain was determined with and without blocking by the COX-2 selective inhibitor *N*-[2-(cyclohexyloxy)-4-nitrophenyl]methanesulfonamide (NS398). Interestingly, [^11^C]**6** appeared to bind specifically to brain regions which were known to possess highest expression levels of COX-2. Furthermore, heterogeneous uptake in the brain was not observed after NS398 administration. The brain distribution of [^11^C]**6** was measured also in herpes simplex virus infected animals which show intense inflammatory responses linked with high expression of COX-2 in the brain. This revealed a moderate but not significant increase of tracer uptake compared to healthy control animals. The rather homogenous uptake was confirmed by small animal PET imaging in parallel studies with [^11^C]PK11195, a widely used PET tracer which targets microglia activation during neuroinflammation [45], and corresponded to activated microglia. The uptake of [^11^C]**6** in inflamed tissue was also investigated by a sterile inflammation model using turpentine injection into the muscle to induce inflammatory response. Here, also no enhanced tracer uptake into inflamed tissue was found and a blocking of uptake was only observed by using the nonselective COX inhibitor indomethacin but not by NS398. So finally, [^11^C]**6** was not able to detect unambiguously the COX-2 expression in the tested inflammation models.

**Scheme 11:**
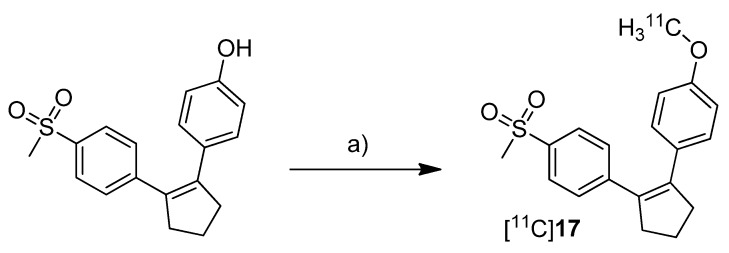
Synthesis of [^11^C]**17**.

Three years after the synthesis of [^18^F]**1** and [^18^F]**2**, Wuest *et al.* presented the synthesis and the *in vitro* and *in vivo* evaluation of 1-[^11^C]methoxy-4-(2-(4-(methylsulfonyl)phenyl)cyclopent-1-en-1-yl)benzene ([^11^C]**17**, Scheme 11), a COX-2 selective inhibitor with an IC_50_ value of 0.005 µM and 9.9 µM for COX-2 and COX-1, respectively [30]. 

*Reagents and conditions*: (**a**) [^11^C]CH_3_I, NaOH, DMF, 60 °C, 3 min.

**Synthesis:** After [^11^C]methyl iodide was trapped in a mixture of DMF and 5 M NaOH at −20 °C the appropriate hydroxy precursor was radiolabeled by *O*-[^11^C]methylation at 60 °C for 3 min, purified by semi-preparative HPLC and formulated for the following experiments. In this way, [^11^C]**17** was synthesized in 19% decay corrected radiochemical yield (based on [^11^C]carbon dioxide) with a specific activity of 20–25 GBq/µmol and a radiochemical purity > 95% within 35 min. ***In vitro*:** At first, [^11^C]**17** was investigated in cell uptake studies with cell lines having a low baseline expression pattern of COX-2, namely unstimulated human (THP-1) and murine (RAW 264.7) monocytes/macrophages and quiescent murine fibroblasts (NIH 3T3). In a second experimental block cell lines with upregulated COX-2 expression were used, as like as human tumor cell lines (squamous cell carcinoma (FaDu) and colorectal adenocarcinoma (HT29)), mouse preadipocytes (3T3 L1) and 12-*O*-tetradecanoylphorbol-13-acetate (TPA)-stimulated THP-1 and RAW 264.7 cells. In these studies, [^11^C]**17** showed specific uptake in COX-2 expressing cells which could also be blocked by pre-incubation with the non-radioactive reference. ***In vivo*:** Biodistribution studies in Wistar rats revealed a high uptake of [^11^C]**17** in the liver, pancreas and white adipose tissue together with high initial brain uptake and elimination by hepatobiliary route. The finding that [^11^C]**17** penetrates the BBB and accumulated in the adipose tissue was found to be consistent with the high calculated lipophilicity of the tracer of log *P* = 4.2 (ACDLab). Metabolite analysis revealed a fast metabolization to a single more polar compound so that after 60 min only 2% and 18% of intact [^11^C]**17** were found in mouse and rat plasma, respectively. Compound [^11^C]**17** was furthermore tested in nude mice bearing HT-29 xenograft tumors. Although an uptake of [^11^C]**17** in the tumor tissue was clearly visible, this could not be blocked by pre-injection with the non-radioactive reference, pointing to a more non-specific uptake.

**Scheme 12:**
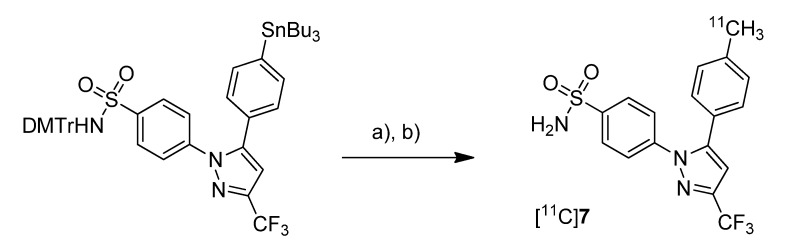
Synthesis of [^11^C]**7**.

Three years after the synthesis of [^11^C]celecoxib ([^11^C]**7**) by Prabhakaran *et al.* [61], de Vries *et al.* presented their own studies with [^11^C]**7** (Scheme 12) to determine whether celecoxib affects the P-gp-mediated drug efflux [67]. Within this work, the brain distribution of [^11^C]**7** was determined by *ex vivo* analysis and microPET imaging.

*Reagents and conditions*: (**a**) Pd_2_(dba)_3_, P(*o*-tolyl)_3_, [^11^C]CH_3_I, DMF, 130 °C, 5 min; (**b**) 200 µL TFA in 0.3 mL DMF, 130 °C, 5 min.

**Synthesis:** The synthesis of [^11^C]**7** was based on the previously described protocol [61] with several modifications including higher concentrations of the reactants and a modified work up after the deprotection step. In this way, the radiotracer was synthesized in 10–15% radiochemical yield with an average specific activity of 19 GBq/µmol. ***In vivo*/*Ex vivo*:** To determine whether celecoxib is a P-gp substrate, Male Wistar rats were treated with 50 mg/kg CsA, a Pgp-inhibitor, and the brain uptake of [^11^C]**7** was measured whereby untreated controls served as references. *Ex vivo* biodistribution studies revealed that the highest accumulation of [^11^C]**7** was found in blood cells, liver, spleen, pancreas and lung as well. Generally, the uptake in these organs was higher than in plasma indicating a good distribution of the radiotracer. A relatively high uptake into the brain demonstrated that [^11^C]**7** is able to penetrate the blood brain barrier (BBB). It was pointed out that this is in accordance with findings from Prabhakaran *et al.* [44] who showed that [^18^F]**16**, the fluorine-18 analog, has entered the brain. However, the radioactivity within the brain was homogenously distributed and did not reflect the distribution of COX-2 probably due to nonspecific binding. Further tests with inhibition by CsA revealed that celecoxib is a poor or even no substrate of P-gp. It was also shown that celecoxib is a poor inhibitor of P-gp in the BBB.

#### 2009

In 2009, Takashima-Hirano *et al.* presented their results regarding the synthesis of [^11^C]**7** by *C*-[^11^C]methylation of an organoboron precursor and PET imaging in rat brain in a poster presentation [68]. The radiosynthesis was presented in detail within a publication in 2011. *In vivo*: [^11^C]**7** showed uptake in the cerebral hemisphere where COX-2 expression was induced by unilateral cortical spreading depression. This uptake could be blocked by administration of unlabeled NS-398. 

#### 2010

**Scheme 13:**
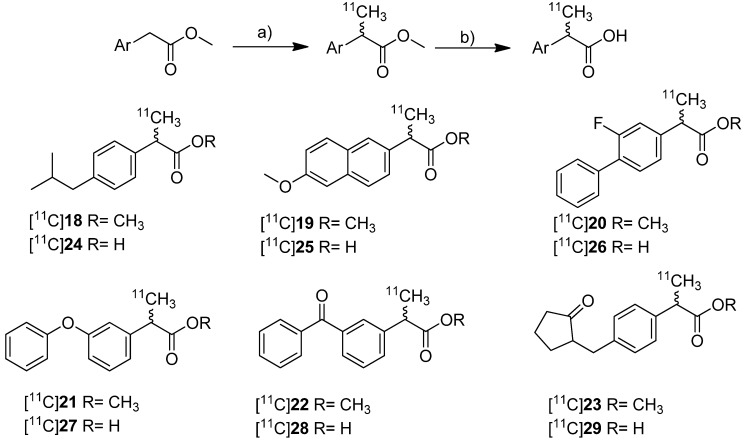
Synthesis of [^11^C]**18**–**29**.

In 2010, synthesis and *in vivo* evaluation of six carbon-11-labeled NSAIDs and their appropriate prodrugs was reported. Takashima-Hirano *et al.* presented a general method to synthesize carbon-11-labeled 2-arylpropionic acids as unselective COX inhibitors and its esters as appropriate prodrugs [45]. In this work, a set of carbon-11-labeled analogs of ibuprofen ([^11^C]**24**), naproxen ([^11^C]**25**), flurbiprofen ([^11^C]**26**), fenoprofen ([^11^C]**27**), ketoprofen ([^11^C]**28**) and loxoprofen ([^11^C]**29**) was synthesized (Scheme 13) which shared this common structural motif of NSAIDs. The COX-inhibitory potency of the carboxylic acid derivatives was already described and exhibits no selectivity or rather selectivity towards COX-1 [69,70].

*Reagents and conditions*: (**a**) [^11^C]CH_3_I, NaH, DMF, 30 °C, 2 min; (**b**) for [^11^C]**24**–**29**: 2 M NaOH, DMF, 50 °C, 1 min.

**Synthesis:** The synthesis of [^11^C]**18**–**23** was performed by *C*-[^11^C]methylation of the corresponding enolates which were generated *in situ* by deprotonation of the benzylic position with NaH and subsequent reaction with [^11^C]methyl iodide in DMF at 30 °C. This gave the methyl propionic acid esters [^11^C]**18**–**23** after semi-preparative HPLC in 26–72% decay corrected radiochemical yield (based on [^11^C]methyl iodide) with specific activities between 20 ± 9 GBq/µmol for [^11^C]**23** and 47 ± 11 GBq/µmol for [^11^C]**22** within 28–41 min total synthesis time. Compounds [^11^C]**18**, [^11^C]**19**, [^11^C]**22**, and [^11^C]**23** were sensitive towards radiolysis but this tendency could be prevented by addition of ascorbic acid and a radiosynthesis with an activity level less than 15 GBq [^11^C]methyl iodide. The carbon-11-labeled carboxylic acids [^11^C]**24**–**29** were synthesized in an one pot reaction by hydrolysis of the esters [^11^C]**18**–**23** after *C*-[^11^C]methylation by addition of 2 M NaOH solution and subsequent heating to 50 °C for 1 min. Purification by semi-preparative HPLC gave [^11^C]**24**–**29** in 29–72% decay corrected radiochemical yield (based on [^11^C]methyl iodide) with specific activity between 20 ± 5 GBq/µmol for [^11^C]**27** and 31 ± 12 GBq/µmol for [^11^C]**23** within 28–42 min total synthesis time. In contrast to the esters, the carboxylic acids were found to be stable towards radiolysis. *In vivo*: [^11^C]**22** and [^11^C]**28** were evaluated in male Sprague-Dawley rats. Small animal PET studies of rats with lipopolysaccharide (LPS)-induced inflammation in the left striatum revealed a high accumulation in the LPS injection side and surrounding tissue after administration of [^11^C]**22**. The authors found that [^11^C]**22** showed a similar uptake compared to [^11^C]PK11195 but with a better ratio regarding inflammatory region to background. In contrast to [^11^C]**22**, [^11^C]**28** showed only low uptake into the brain. Metabolite analysis of [^11^C]**22** in blood of normal rats revealed a fast hydrolysis to the biologically active form [^11^C]**28** and a lower hydrolysis rate in brain. However, after uptake of the ester in the brain still more than 90% was hydrolyzed within 5 min into the acid derivative [^11^C]**28** what was expected by the authors to be fast enough for detection of [^11^C]**28** in the brain. After that time point, significant differences between inflamed region and background were observed. Further PET studies with [^11^C]**18**–**23** showed that all compounds with exception of [^11^C]**18** were accumulated in the LPS induced-inflammatory region of the brain with the highest accumulation of [^11^C]**22** and best ratio of inflamed tissue to contralaterally lesioned area for [^11^C]**19** and [^11^C]**23**. Blocking studies with [^11^C]**22** were performed by simultaneous injection of the non-radioactive reference and revealed a significantly reduced accumulation of radioactivity in the LPS-induced inflammatory region.

**Scheme 14:**
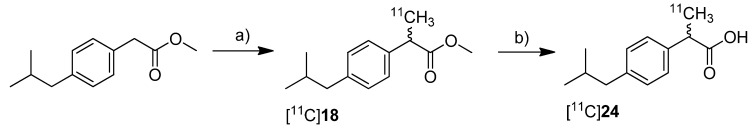
Synthesis of [^11^C]**18** and [^11^C]**24**.

Kato *et al.* presented the synthesis of [^11^C]ibuprofen ([^11^C]**24**) as an example for the developed TBAF promoted synthesis of 2-arylpropionic acids [71] (Scheme 14). As valuable aspect in comparison to the procedure developed by Takashima-Hirano *et al.* [45], the authors point out that the use of TBAF instead of NaH allows performing the automated labeling reaction under homogenous conditions. **Synthesis:** Starting from the appropriate phenyl acetic acid ester precursor and deprotonation of the benzylic position by TBAF in THF at room temperature, [^11^C]**18** was synthesized by *C*-[^11^C]methylation with [^11^C]methyl iodide. This yielded [^11^C]**18** in 89.3 ± 3.3% decay corrected radiochemical yield determined by HPLC in the first step. As reported by Takashima-Hirano *et al.* [45] the authors observed as well significant radiolysis of [^11^C]**18** when the synthesis started with > 15 GBq of [^11^C]carbon dioxide. The radiosynthesis of [^11^C]**24** was performed in an one pot reaction by *C*-[^11^C]methylation followed by addition of 1 M NaOH solution and methanol and subsequent alkaline hydrolysis at 80 °C for 3 min. In this way, [^11^C]**24** was synthesized in 77.3 ± 4.9% decay corrected radiochemical yield determined by HPLC in two steps. Starting from 11.1 GBq of [^11^C]carbon dioxide, the remote-controlled procedure gave > 1 GBq of [^11^C]**24** after semi-preparative HPLC within 30 min total synthesis time. ***In vitro/in vivo*:** Further information about *in vivo* evaluation of the enantiomerically pure radiotracer was presented one year later by Kikuchi *et al.* [72]. 

*Reagents and conditions*: (**a**) [^11^C]CH_3_I, TBAF, THF, r.t. 30 s; (**b**) 1 M NaOH/MeOH 1:1, 80 °C, 3 min.

#### 2011

In 2011, the synthesis of two fluorine-18-labeled and fifteen carbon-11-labeled COX-2 inhibitors and, for a part of them, the *in vitro/in vivo* evaluation as well the *in vivo* evaluation of the carbon-11-labeled prodrug of ketoprofen and carbon-11-labeled (*R*)- and (*S*)-ibuprofen was presented.

**Scheme 15:**
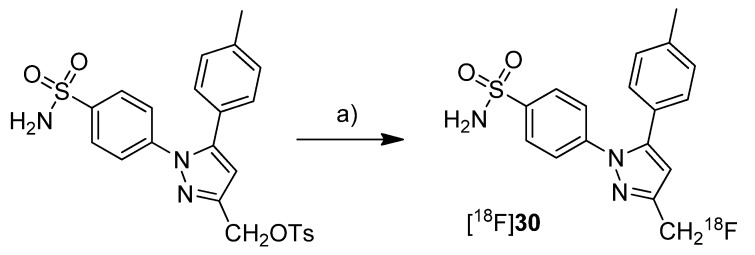
Synthesis of [^18^F]**30**.

*Reagents and conditions:* (**a**) [^18^F]KF, K_222_, DMSO, microwave, 165 °C, 3 min.

**Scheme 16:**
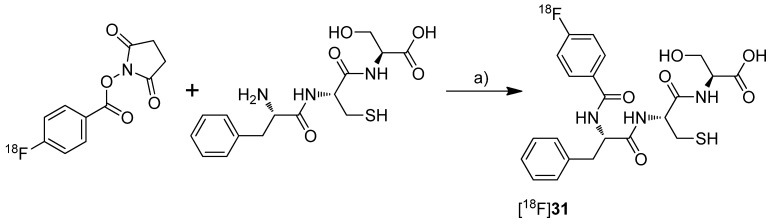
Synthesis of [^18^F]**31**.

Uddin *et al.* presented the synthesis and *in vivo* evaluation of 4-(3-([^18^F]fluoromethyl)-5-(*p*-tolyl)-1*H*-pyrazol-1-yl)benzenesulfonamide ([^18^F]**30**, Scheme 15), a fluorine-18-labeled celecoxib derivative [48]. This compound was selected from a set of synthesized celecoxib and indomethacin derivatives due to its high and selective COX-2 inhibition potency. The non-radioactive compound 30 revealed *in vitro* an IC_50_ value of 0.16 µM against purified human COX-2, 0.08 µM against intact COX-2 expressing RAW 264.7 cells and > 4 µM against ovine COX-1. **Synthesis**: [^18^F]**30** was synthesized by fluorine-18-for-tosylate exchange in a microwave assisted reaction. For this, the tosylate precursor in DMSO was added to the dried [^18^F]KF-kryptofix complex followed by heating to 165 °C and sequential additional heating by microwaves. This gave [^18^F]**30** after semi-preparative HPLC in 25% radiochemical yield and in a radiochemical purity > 99%. *In vivo*: For the evaluation of [^18^F]**30**, male Sprague-Dawley rats with a carrageenan-induced inflammation in the rear right paw were used. 1.5 h after injection of [^18^F]**30**, the animals were studied by small animal PET. The uptake in the inflamed paw was higher compared to the contralateral, non-inflamed paw and this enhanced uptake could be blocked by administration of celecoxib immediately prior to [^18^F]**30** injection. These results were confirmed by post mortem analysis which revealed a 1.53 ± 0.5-fold (n = 5) higher amount of radioactivity in the inflamed compared to the non-inflamed paw. The specificity for COX-2 was examined by intraperitoneal (i.p.) administration of [^18^F]**30** to wild-type and COX-2 null mice. In COX-2 null mice no statistical significant differences were observed between [^18^F]**30** uptake into the carrageenan-induced inflamed paw compared to the non-inflamed paw. In contrast, wild-type mice showed a significantly higher uptake in the inflamed tissue. The stability of the tracer was tested by i.p. injection of the non-radioactive reference 30. The amount of defluorinated metabolite after 2 hours was detected by LC-ESI-MS in extracts of the pad tissue after euthanasia what revealed 8.8 ± 5% (n = 3) of defluorinated metabolite. [^18^F]**30** was furthermore tested in tumor xenograft bearing female nude mice. For this, COX-2 negative human colorectal carcinoma (HCT116) and COX-2-expressing human head and neck squamous cell carcinoma (1483 HNSCC) tumor xenografts were allowed to grow in the right and left hip of the mice, respectively. Small animal PET studies revealed a significant uptake of [^18^F]**30** in the COX-2 expressing tumor which was approximately 3-fold higher compared to the uptake in COX-2 null tumor tissue as determined by *ex vivo* distribution analysis. Furthermore, the specific uptake of [^18^F]**30** was demonstrated by i.p. administration of celecoxib and a vehicle immediately prior to radiotracer injection. In comparison, the tumor-to-muscle ratio was decreased from 2.94 ± 0.36 (n = 5) to 1.01 ± 0.16 (n = 4) when celecoxib was administrated instead of the vehicle. With this work, Uddin *et al.* furnished proof for the first time that COX-2 can be visualized selectively and specific with a fluorine-18-labeled COX-2 inhibitor by means of PET.Al-Hourani *et al.* presented in a poster presentation the synthesis and *in vitro* results of the fluorine-18-labeled tripeptide *N*-(4-[^18^F]Fluorobenzoyl)-phenylalanyl-cysteyl-serine ([^18^F]**31**, Scheme 16) possessing an IC_50_ for COX-2 of 13 µM and for COX-1 of 27 µM [73]. The synthesis and evaluation of this and other but less potent non-radiolabeled fluorobenzoylated di- and tripeptide derivatives was published recently [74]. **Synthesis**: [^18^F]**31** was synthesized by labeling the tripeptide precursor with *N*-succinimidyl 4-[^18^F]fluorobenzoate ([^18^F]SFB) and subsequent semi-preparative HPLC in 5–8% radiochemical yield (based on [^18^F]SFB) with a radiochemical purity exceeding 95% within 70 min. *In vitro*: [^18^F]**31** uptake was tested in COX-2 expressing Ward colon tumor cells but this revealed only low uptake which could not be blocked by celecoxib. 

*Reagents and conditions:* (**a**) Borate buffer (pH = 8.3), 60 °C, 30 min.

**Scheme 17:**
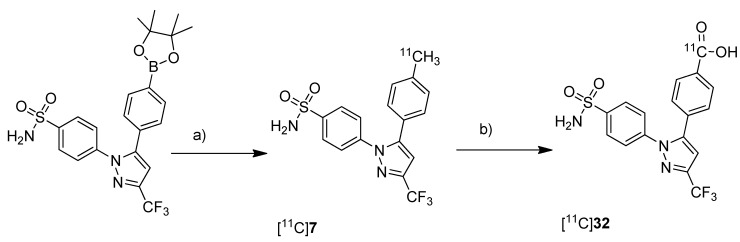
Synthesis of [^11^C]**7** and [^11^C]**32**.

Takashima-Hirano *et al.* presented the synthesis and *in vivo* evaluation of carbon-11-labeled celecoxib [^11^C]**7** (Scheme 7) and its major metabolite 4-(1-(4-sulfamoylphenyl)-3-(trifluoromethyl)-1*H*-pyrazol-5-yl)benzoic acid [^11^C]**32** (Scheme 17) with the aim to examine the function of drug transporters for hepatobiliary excretion [75]. **Synthesis:** [^11^C]**7** was synthesized in an one step reaction starting from an organoboron precursor by reaction with [^11^C]methyl iodide in the presence of Pd_2_(dba)_3_, P(*o*-tolyl)_3_ and K_2_CO_3_ in DMF for 5 min at 130 °C. This yielded compound [^11^C]**7** in 63 ± 23% (n = 7) decay corrected radiochemical yield with a specific activity of 84 ± 23 GBq/µmol within 30 min synthesis time from end of bombardment. [^11^C]**32** was synthesized by subsequent oxidation of [^11^C]**7** with KMnO_4_ in 0.2 M NaOH under microwave irradiation. In this way, [^11^C]**32** was synthesized in 55 ± 9% (n = 3) decay corrected radiochemical yield (based on [^11^C]**7**) with a specific activity of 39 ± 4 GBq/µmol after 50 min synthesis time from end of bombardement. ***In vivo*/*Ex vivo*:** Both radiotracers have been evaluated with focus on their hepatobiliary excretion using male Sprague-Dawley rats. After initial uptake in the liver, [^11^C]**7** moved to the intestine. Metabolite analysis revealed that [^11^C]**7** was found in blood and liver with only small amounts of metabolites but that it was completely degraded to the carboxylic acid metabolite [^11^C]**32** in the bile. When [^11^C]**32** was injected, no further metabolites were detected and radioactivity was rapidly excreted by hepatobiliary and renal routes.

*Reagents and conditions*: (**a**) [^11^C]CH_3_I, Pd_2_(dba)_3_, P(*o*-tolyl)_3_, 65 °C, DMF, 4 min; (**b**) KMnO_4_, 0.2 M NaOH, microwave, 140 °C, 5 min.

**Scheme 18:**
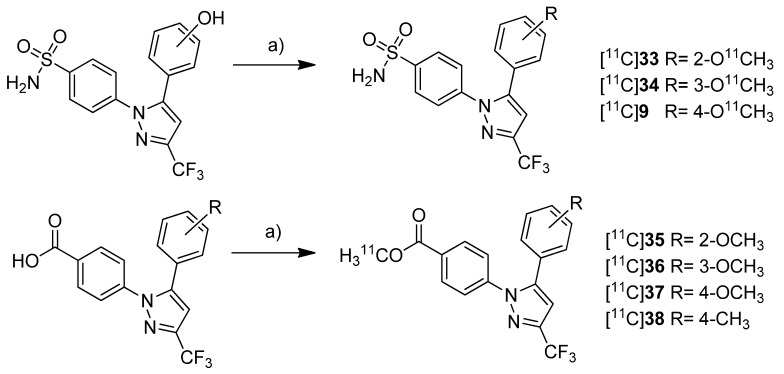
Synthesis of [^11^C]**9 **and [^11^C]**33**–**38**.

Gao *et al.* presented the synthesis of one known ([^11^C]**9**) and six novel ([^11^C]**33**–**38**) COX-2 inhibitors labeled with carbon-11 and the *in vitro* evaluation of the corresponding non-radioactive reference compounds [46] (Scheme 18). The IC_50_ values for COX-2 of the non-radioactive analogs 4-(5-(2-methoxyphenyl)-3-(trifluoromethyl)-1*H*-pyrazol-1-yl)benzenesulfonamide (**33**) and 4-(5-(4-methoxyphenyl)-3-(trifluoromethyl)-1*H*-pyrazol-1-yl)benzenesulfonamide (**9**) were reported earlier to be 290 nM and 9 nM [62]. 

*Reagents and conditions*: (**a**) [^11^C]CH_3_OTf, 2 M NaOH, MeCN, 80 °C, 3 min.

The non-radioactive references were tested in a cell proliferation assay with a MDA-MB-435 tumor cell line. A statistical significant difference for the inhibition of proliferation at different concentration was found for compounds **35** and **37**. **35** and **37** showed inhibition of cell proliferation in a range of 160–320 nM and at 160 nM, respectively. All other compounds did not significantly inhibit cell proliferation ranging between 20 and 1280 nM. **Synthesis:** All compounds were radiolabeled by *O*-[^11^C]methylation of the corresponding phenolic hydroxy or carboxylic acid precursor. In brief, [^11^C]methyl triflate was prepared as methylating agent from [^11^C]carbon dioxide and reacted with the precursor in acetonitrile basified with 2 N NaOH. This yielded the *O*-[^11^C]methylated ethers and esters after purification by solid phase extraction within 23 min in decay corrected radiochemical yields of 52 ± 2% (n = 5) and 57 ± 3% (n = 5) (based on [^11^C]carbon dioxide), respectively. The specific activity was determined to be 277.5 ± 92.5 GBq/µmol (n = 5) and the radiochemical purity was > 99%. ***In vitro*/*In vivo*:** No further *in vitro* or *in vivo* evaluation was presented.

**Scheme 19:**
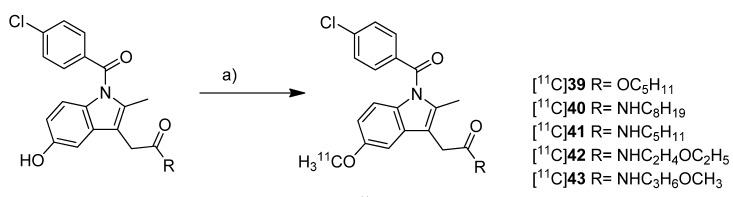
Synthesis of [^11^C]**39**–**43**.

*Reagents and conditions*: (**a**) [^11^C]CH_3_OTf, NaOH, acetone, r.t.

Yamamoto *et al.* presented five years after the synthesis of [^11^C]**13**–**15** the synthesis of five carbon-11-labeled indomethacin derivatives ([^11^C]**39**–**43**, Scheme 19) as well as their *in vitro* and *in vivo* evaluation [35]. The non-radioactive analogs pentyl 2-(1-(4-chlorobenzoyl)-5-methoxy-2-methyl-1*H*-indol-3-yl)acetate (**39**) and 2-(1-(4-chlorobenzoyl)-5-methoxy-2-methyl-1*H*-indol-3-yl)-*N*-octyl-acetamide (**40**) have been described earlier as potent COX-2 inhibitors [76] and served as lead structure for the development of the presented compounds. The non-radioactive compounds were tested in an *in vitro* COX assay which revealed that compound 2-(1-(4-chlorobenzoyl)-5-methoxy-2-methyl-1*H*-indol-3-yl)-*N*-pentylacetamide (**41**) had the highest affinity and selectivity for COX-2 with an IC_50_ value for COX-2 of 0.039 µM and an IC_50_ value for COX-1 of more than 100 µM. The other compounds were selective COX-2 inhibitors, too, but with IC_50_ values for COX-2 in the range of 0.2–2.3 µM. **Synthesis:** All compounds were radiolabeled by *O*-[^11^C]methylation from their corresponding desmethyl precursor. This was realized in detail by reaction with [^11^C]methyl triflate in acetone in the presence of NaOH and purification with semi-preparative HPLC. That protocol gave the desired radiotracer in a decay corrected radiochemical yield (based on [^11^C]methyl triflate) in the range of 55 ± 12% in case of [^11^C]**40** to 71 ± 5% (n = 5) in case of [^11^C]**42** within 21-27 min total synthesis time and a radiochemical purity of 93-97%. From [^11^C]**39** to 2-(1-(4-chlorobenzoyl)-5-[^11^C]methoxy-2-methyl-1*H*-indol-3-yl)-*N*-(3-methoxypropyl)acetamide ([^11^C]**43**)**,** specific activity increased in the range of 22-331 GBq/µmol and lipophilicity determined as log *P*_7.4_ decreased from 3.94 to 1.98. ***In vivo*/*Ex vivo*:** [^11^C]**39**–**43 ** were evaluated *in vivo* in male ddY mice. Compounds [^11^C]**40**–**43** showed a relatively fast blood clearance with a half-life of 1.3–3.8 min in contrast to compound [^11^C]**39** with a half-life of 12.3 min. The radioactivity initially accumulated for all radiotracers in the liver and a peak concentration was observed in the small intestine reflecting predominant hepatobiliary clearance of the tracers or their metabolites, respectively. The brain uptake of all compounds was low and inversely related with the lipophilicity what was expected due to the increased plasma protein binding. Metabolite analysis revealed the low stability of the radiotracers *in vivo* due to hydrolysis of the ester and amide functionality and further catabolic reactions. In case of [^11^C]**39** no intact parent compound was found in plasma after 30 min and for compounds [^11^C]**40**–**43 **the amount was 14–32%. In the brain only 9–26% of intact parent compound was detected as well. Blocking studies with either celecoxib, NS-398 or unlabeled compound did not inhibit brain uptake and did not influence brain-to-blood ratio what indicates non-specific binding of the tracers *in vivo* except for [^11^C]**41** what showed a small fraction of displaceable binding. To find out whether P-gp-mediated drug efflux played a role for the low brain uptake, all radiotracers were evaluated after pretreatment with CsA, a P-gp inhibitor. After pretreatment, a slightly increased uptake in the brain and increased brain-to-blood ratio was observed indicating a possible influence of P-gp in the transport of the radiotracer or an altered cytochrome P450-mediated metabolism caused by CsA.

**Scheme 20:**
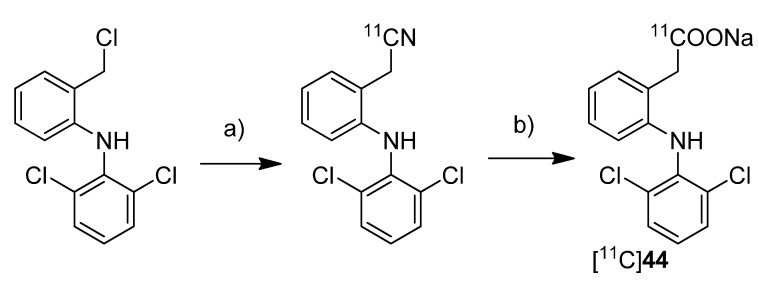
Synthesis of [^11^C]**44**.

To investigate the transdermal penetration of a topical applied drug by means of PET, Petroni *et al.* synthesized carbon-11-labeled diclofenac ([^11^C]**44**, Scheme 20) and evaluated the tracer by means of PET after topical formulation [40]. 

*Reagents and conditions*: (**a**) [^11^C]NaCN, DMSO, 110 °C, 10 min; (**b**) 10 M NaOH, 30% H_2_O_2_, DMSO/H_2_O, 135 °C, 10 min.

**Synthesis:** For the synthesis sodium [^11^C]cyanide was used which was produced starting from [^11^C]carbon dioxide by reduction to [^11^C]methane and subsequent reaction on platinum in the presence of anhydrous ammonia. By reacting the precursor 2-[(2,6-dichlorophenyl)amino]benzyl chloride with sodium [^11^C]cyanide in DMSO at 110 °C, the corresponding nitrile was formed in 84% radiochemical yield as determined by HPLC. Subsequently the hydrolysis was performed by addition of 1 M NaOH and H_2_O_2_ and heating to 135 °C. This gave [^11^C]**44** in 43 ± 4% (n = 20) radiochemical yield as determined by HPLC. After preparative HPLC, [^11^C]**44** was obtained in 97 ± 4% (n = 20) radiochemical purity and formulated for topical application with a non-pressurized sprayer. For this, the dried product was mixed with the diclofenac preparation of the manufacturer. The preliminary evaluation of the transdermal penetration of [^11^C]**44** by means of PET showed that [^11^C]**44** rapidly penetrated the keratinized layer of the skin and, hence, showed that PET is a valuable tool to explore transdermal absorption of active ingredients in topical formulations.

**Figure 1:**
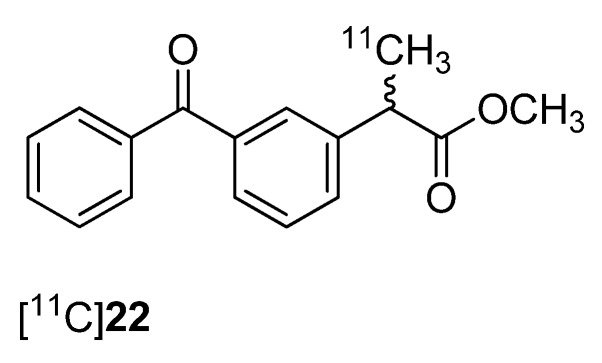
Structure of [^11^C]**22**.

One year after Takashima-Hirano *et al.* presented the synthesis of [^11^C]ketoprofen methyl ester ([^11^C]**22**, Figure 1) [45], Shukuri *et al.* published the results of further biological evaluations of this compound [36]. **Synthesis:** [^11^C]**22** was synthesized as previously reported [45]. *In vivo*/*Ex vivo*: The selectivity of [^11^C]**22** was investigated with male mice homozygous deficient for COX-1 (COX-1^−/−^) and COX-2 (COX-2^−/−^) and their wild-type littermate controls. *Ex vivo* autoradiography revealed a high accumulation in the brain of wild-type mice and COX-2^−/−^ mice and a significantly decreased uptake in COX-1^−/−^ mice showing the COX-1 selectivity of [^11^C]**22** in the brain. Further studies were performed with male Sprague-Dawley rats with LPS-induced or quinoline acid-induced neuroinflammation. PET studies revealed that [^11^C]**22** showed significant time dependent differences in the uptake to the inflamed regions of the brain which reached its maximum when the tracer was administrated 6 h after LPS injection. The appearance of activated microglia and macrophages was correlated to [^11^C]**22** uptake and the expression of COX-1 but not COX-2 in these cells what was shown by immunostaining using IgG antibodies for COX-2 and COX-1. Similar results were obtained regarding the uptake of [^11^C]**22** in the inflammatory regions as well as correlation to microglia and macrophage appearance when quinoline acid was used to induce excitotoxic neurodegeneration in the brain. The authors concluded that these results support the crucial proinflammatory role of COX-1 in neuroinflammatory processes. 

**Scheme 21:**
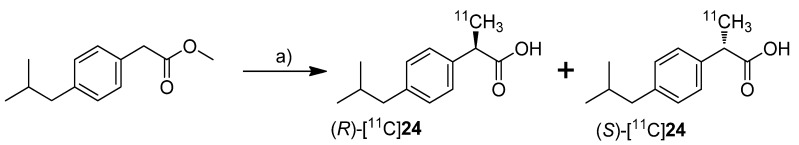
Synthesis of (*R*)-[^11^C]**24** and (*S*)-[^11^C]**24**.

One year after Kato *et al.* [71] and Takashima-Hirano *et al .* [45] have published the synthesis of [^11^C]**24**, Kukushi *et al.* presented the DMSO assisted synthesis, chiral separation and preliminary *in vivo* evaluation of the (*R*)- and (*S*)-enantiomers of [^11^C]**24** [72] (Scheme 21). 

*Reagents and conditions:* (**a**) i: [^11^C]CH_3_I, TBAF, DMSO, r.t. 30 s; ii: 1 M NaOH, 60 °C, 2 min; iii: chiral semi-preparative HPLC.

**Synthesis:** The synthesis procedure was optimized regarding the solvent to suppress the radiolysis of the tracer. The improved procedure using lower amounts of the precursor and DMSO as radical scavenging solvent instead of THF gave [^11^C]**18** in 93 ± 1%, and [^11^C]**24** in 92 ± 3% (in two steps) decay corrected radiochemical yield as determined by HPLC. The chiral semi-preparative HPLC was conducted subsequently to the ester hydrolysis using a gradient of acetonitrile/phosphate buffer on a RP-CHIRALPAC OJ-RH column (DAICEL). In this way, in one synthesis more than 740 MBq of each enantiomer (*R*)-[^11^C]**24** and (*S*)-[^11^C]**24** was isolated (>3% decay uncorrected radiochemical yield based on [^11^C]carbon dioxide) with an specific activity of 66–289 GBq/µmol and radiochemical purity > 99%. The enantiomeric purity was > 95% and 90% for the (*R*)- and (*S*)-isomer, respectively. ***In vivo*:** (*R*)-[^11^C]**24** and (*S*)-[^11^C]**24** were tested in male mice using an anti-collagen induced arthritis model. In comparison to untreated mice, the accumulation of both enantiomers was more than twofold increased in arthritic joints but no differences were observed between the two isomers. For that and the fact that only the (*S*)-enantiomer exhibits COX-inhibition activity, the authors concluded that the accumulation mechanism of [^11^C]**24** in the arthritic joint was independent of COX overexpression. 

#### 2012

In 2012, the synthesis of five new fluorine-18-labeled COX-2 inhibitors was presented, two of them with *in vivo* evaluation.

**Scheme 22:**
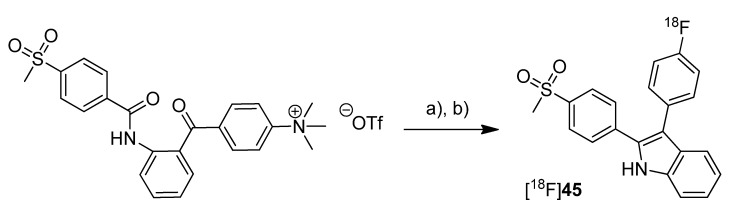
Synthesis of [^18^F]**45**.

In 2012, our group published the automated synthesis and *in vitro* and *in vivo* evaluation of 3-(4-[^18^F]fluorophenyl)-2-(4-(methylsulfonyl)phenyl)-1*H*-indole ([^18^F]**45**) whose radiosynthesis via McMurry cyclization as a new radiolabeling approach has been presented as conference contribution [77] one year earlier [31] (Scheme 22). The non-radioactive reference **45** was previously described as highly potent and selective COX-2 inhibitor with an IC_50_ of 0.02 nM for COX-2 and >10 µM for COX-1 [66]. By a fluorescence-based assay with the purified enzymes, the IC_50_ value of **45** was determined to be 1.2 µM and 6.6 µM for COX-2 and COX-1, respectively. Furthermore, the COX-inhibition potency was verified by an LC-MS/MS based method determining the prostanoid levels in tumor cells and the inhibition of prostanoid synthesis after incubation with the non-radioactive reference compound. 

*Reagents and conditions*: (**a**) [^18^F]KF,K_222_, MeCN, 110 °C, 15 min; (**b**) TiCl_4_, Zn, THF, 90 °C, 15 min.

**Synthesis:** [^18^F]**45 **was synthesized in a two-step procedure which involved the labeling of the activated precursor molecule via [^18^F]fluorine-for-trimethylammonium exchange and was followed by the McMurry ring closure reaction. Different reaction conditions were tested and an optimized protocol for an automated radiosynthesis was presented. Reacting the dried [^18^F]fluoride-kryptofix complex with the precursor in acetonitrile at 110 °C for 15 min, removal of the solvent and dissolving in THF, and final cyclization in the presence of TiCl_4_ and Zn at 90 °C for 15 min formed [^18^F]**45**. A further solvent exchange to acetonitrile/water, filtration, semi-preparative HPLC and formulation gave [^18^F]**45** in 10% decay corrected radiochemical yield with a specific activity of 74–91 GBq/µmol within 80 min total synthesis time. The log D_oct7.4_ of [^18^F]**45** was determined to be 1.2 ± 0.2. **In vitro:** Cell uptake studies of [^18^F]**45** were performed with several cell lines and models which were characterized regarding their COX expression pattern by Western blot analysis. As cell lines with low baseline COX-2 expression served unstimulated human monocytes (THP-1) and the melanoma cell line A375. TPA-stimulated human monocytes (THP-1) and a number of human tumor cell lines—FaDu, HT-29, and A2058 (melanoma)—were used as cell lines with upregulated COX-2 expression. The uptake studies were performed with monolayer cultures and, for selected cell lines, in suspension cultures (unstimulated THP-1). Furthermore, cell uptake experiments were performed in multicellular tumor spheroids with a diameter of 300 µm and 600 µm (HT-29). The uptake was consistent with the COX-1/COX-2 selectivity of [^18^F]**45** and could be blocked by preincubation with the nonradioactive reference. Furthermore, hypoxia-induced COX-2 expression in large-sized spheroids was characterized by increased and specific uptake of [^18^F]**45** and the hypoxia-specific radiotracer [^18^F]FMISO as well. The possible interaction with the P-gp efflux pump was examined in the presence of the P-gp inhibitor CsA and the calcium blocker verapamil but did not induce any significant effect on the tracer uptake. *in vitro* stability of [^18^F]**45** was found to be high in rat whole blood and plasma with appearance of less than 5% of a more polar metabolite after 2 h. **In vivo:** The *in vivo* stability of [^18^F]**45** was examined in male Wistar-Unilever rats by analysis of arterial blood samples revealing that after 1 h at least 75% of original compound was intact and two more polar metabolites with equal amount were formed. To evaluate the capacity of [^18^F]**45** to detect COX-2 expressing tumors, the tracer was investigated by small animal PET studies in HT-29 tumor-bearing nude mice. Unfortunately, no substantial accumulation of the radiotracer was observed in the tumor tissue, instead the main activity was eliminated by the hepatobiliary route after fast accumulation in the liver. A substantial amount of activity was found in the harderian glands and brown adipose tissue.

**Scheme 23:**
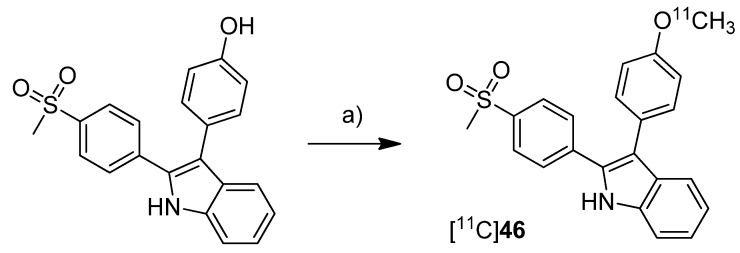
Synthesis of [^11^C]**46**.

In 2012, we have summarized the results regarding the radiosynthesis and cell uptake studies of [^18^F]**45** and another carbon-11-labeled COX-2 inhibitor, 3-(4-[^11^C]methoxyphenyl)-2-(4-(methylsulfonyl)phenyl)-1*H*-indole [^11^C]**46,** as a conference contribution [37] (Scheme 23). **Synthesis:** [^11^C]**46** was synthesized by *O*-[^11^C]methylation from the corresponding desmethyl precursor with [^11^C]methyl iodide in 23% decay corrected radiochemical yield with an specific activity of 79–89 GBq/µmol and 99% radiochemical purity. ***In vitro*:** Comparing cell uptake studies were performed with [^11^C]**45** and [^18^F]**46** using the previously reported [31] cell lines and models. [^11^C]**46** showed like [^18^F]**45** substantially higher uptake in COX-2 upregulated cells.

*Reagents and conditions:* (**a**) [^11^C]CH_3_I, 0.1 M NaOH, acetone, 80 °C, 2 min.

**Scheme 24:**
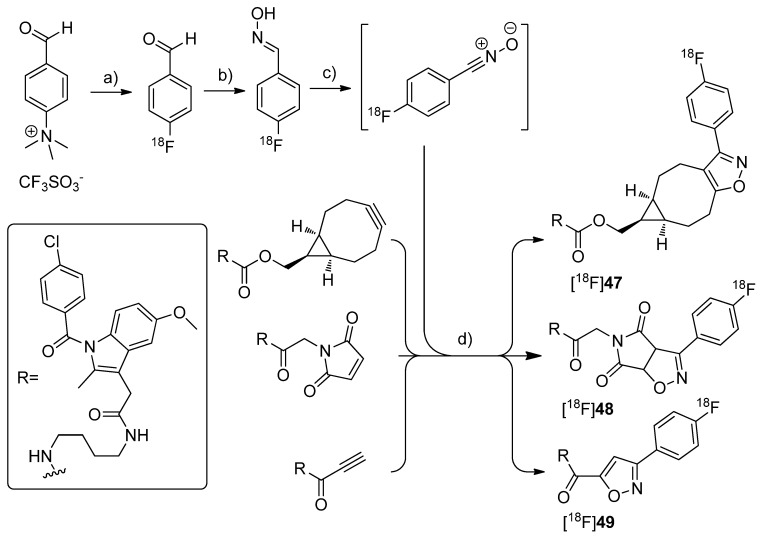
Synthesis of [^18^F]**47**–**49**.

By demonstrating the potential of a new developed fluorine-18-labeling method, the metal-free [3+2]-nitrile oxide/alkyne cycloaddition, Zlatopolskiy *et al.* synthesized three new isoxazole analogs of indomethacin esters [^18^F]**47**–**49** as potential COX-2 specific tracers [78] (Scheme 24). Similar fluorescent-dye labeled derivatives of indomethacin have been described by Uddin *et al.* as the first *in vivo* fluorescent optical imaging agents for COX-2 [79]. 

*Reagents and conditions*: (**a**) [^18^F]KF,K_222_, DMSO, 85 °C, 10 min; (**b**) NH_2_OH·HCl, 1 M NaOH, MeOH, 40 °C, 10 min; (**c**) PIFA in MeOH for [^18^F]**47** and [^18^F]**48**, BAIB in DMF for [^18^F]**49**, 5–10 s; (**d**) MeOH, r.t. for [^18^F]**47**, 40 °C for [^18^F]**48**, 60 °C for [^18^F]**49**, 10 min.

**Synthesis:** [^18^F]**47**–**49** were synthesized in a four step procedure which involved the [3+2]-nitrile oxide/alkyne cycloaddition as final key step. 4-[^18^F]fluorobenzonitrile oxide was synthesized starting from [^18^F]fluoride and the trimethylammonium benzaldehyde precursor to yield [^18^F]fluorobenzaldehyde in the first step in 30-50% radiochemical yield within 50 min synthesis time. Afterwards, [^18^F]fluorobenzaldehyde was converted into the respective aldoxime by reaction with hydroxylamine hydrochloride and NaOH in methanol at 40 °C for 10 min with 92.1 ± 2.4% radiochemical yield (n = 23). To the crude 4-[^18^F]fluorobenzaldoxime (radiochemical purity > 90%) was then added phenyl iodine bis(trifluoroacetate) (PIFA) in case of [^18^F]**47**–**48** and [bis(acetoxy)iodo]benzene (BAIB) in case of [^18^F]**49 ** to generate *in situ* 4-[^18^F]fluorobenzonitrile oxide within 5–10 s. The *in situ* generated nitrile oxide was allowed to react with the appropriate labeling precursor for 10 min at room temperature, 40 °C and 60 °C to yield [^18^F]**47** in 81.4 ± 2.0%, [^18^F]**48** in 54.8 ± 1.3% and [^18^F]**49** in 35.3 ± 4.7% radiochemical yield (n = 3) after preparative HPLC, respectively. Finally, [^18^F]**47**–**49** were formulated in 20% ethanol for further biological experiments. ***In vitro*/*In vivo*:** A biological study is in progress but results are not published yet.

### 2.2. COX-2 Inhibitors Radiolabeled with SPECT Nuclides

In comparison to a number of COX-2 inhibitors radiolabeled with PET isotopes fewer compounds have been radiolabeled with SPECT nuclides. 

#### 2005

**Scheme 25:**
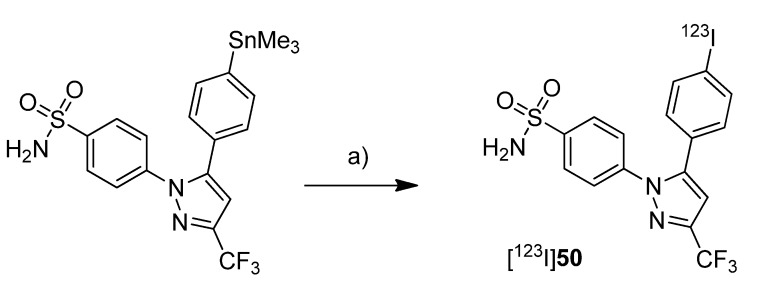
Synthesis of [^123^I]**50**.

In 2005, Kabalka *et al.* presented the synthesis of an iodine-123-labeled 4-iodo analog of celecoxib [52]. **Synthesis:** 4-(5-(4-[^123^I]Iodophenyl)-3-(trifluoromethyl)-1*H*-pyrazol-1-yl)benzene-sulfonamide ([^123^I]**50**) was synthesized by radioiodination of the corresponding trimethylstannylated precursor (Scheme 25). 

*Reagents and conditions*: (**a**) [^123^I] NaI in 0.1% NaOH, CH_3_COOOH, MeOH, r.t., 5 min.

For this, no carrier added sodium [^123^I]iodide was stirred with the precursor in presence of diluted peracetic acid at room temperature. Deactivation with Na_2_S_2_O_3_ and purification by solid phase extraction and thin layer chromatography gave [^123^I]**50** in 90% decay corrected radiochemical yield and >98% radiochemical purity within 15 min total synthesis time. ***In vitro*/*In vivo*:** Further information about *in vitro* or *in vivo* evaluation of the tracer was given in a later publication by Schuller *et al.* [53].

#### 2006

**Scheme 26:**
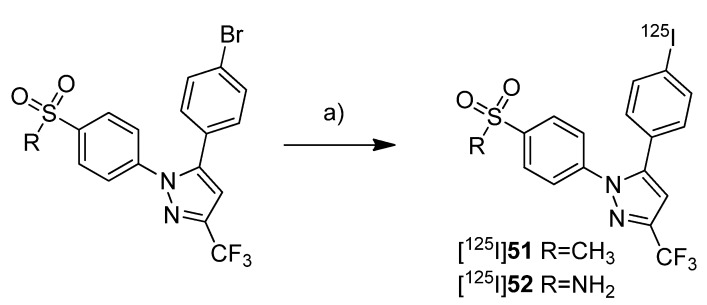
Synthesis of [^125^I]**51 **and [^125^I]**52**.

The synthesis and evaluation of 5-(4-[^125^I]iodophenyl)-1-(4-(methylsulfonyl)phenyl)-3-(trifluoromethyl)-1*H*-pyrazole ([^125^I]**51**) was shown by Kuge *et al.* in a poster presentation [38] and presented in detail with the radiosynthesis and*ex vivo* evaluation of [^125^I]**51 **(Scheme 26) and 4-(5-(4-[^125^I]iodophenyl)-3-(trifluoromethyl)-1*H*-pyrazol-1-yl)benzenesulfonamide ([^125^I]**52**) in 2006 [39] (Scheme 26).

*Reagents and conditions*: (**a**) [^125^I]NaI, (NH_4_)_2_SO_4_, CuSO_4_·5 H_2_O, H_2_O, 140 °C, 2 h.

The COX-inhibitory potency of **51** and **52** was determined with a colorimetric COX inhibitor assay using ovine COX-1 and COX-2 which revealed IC_50_ values for COX-2 of 5.16 µM and 8.2 µM, respectively, and IC_50_ values for COX-1 of > 100 µM. **Synthesis:** The radioiodinated celecoxib analog [^125^I]**52** and its methylsulfonyl substituted derivative [^125^I]**51** were synthesized by iodine-125-for-bromine exchange by reacting the corresponding bromo precursor with sodium [^125^I]iodide in the presence of ammonium sulfate and copper(II) sulfate pentahydrate in water at 140 °C for 2 h. This gave the radiotracers after filtration, semi-preparative HPLC, and formulation in a radiochemical yield of 42% for [^125^I]**51** and 35% for [^125^I]**52** and radiochemical purity of > 95%. *In vivo*/*Ex vivo*: Male Sprague-Dawley rats were used for biodistribution studies. For both compounds high levels of radioactivity were found in the liver and the kidneys but not in the stomach and thyroid, the latter indicates the stability of both compounds. In comparison to [^125^I]**52**, there was for [^125^I]**51** less radioactivity observed in the blood, in the heart and in the lung. Furthermore, [^125^I]**51** showed a higher brain-to-blood ratio of 1.67–3.19 in comparison to 0.36–0.48 for [^125^I]**52**. For compound [^125^I]**51** this was compatible with the known distribution of COX-2. To evaluate the effect of binding to carbonic anhydrase in erythrocytes what is a well-known effect for sulfonamide type inhibitors, the distribution to blood cells was examined for both tracers. [^125^I]**51** showed significantly lower binding to blood cells than [^125^I]**52**. Furthermore, binding of [^125^I]**52** to blood cells decreased by blocking with carbonic anhydrase inhibitors acetazolamide and chlorthalidone, but this was not the case for [^125^I]**51**. 

**Scheme 27:**
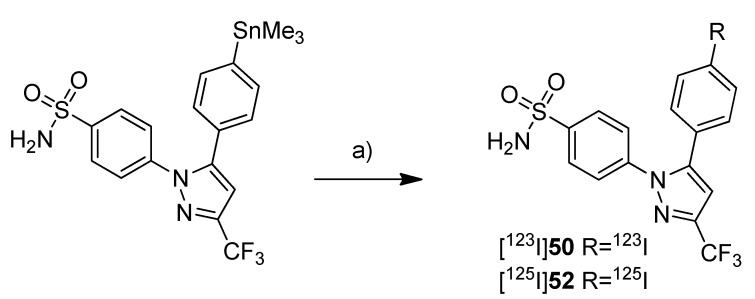
Synthesis of [^123^I]**50 **and [^125^I]**52**.

In 2006, Schuller *et al.* presented the *in vitro* and *in vivo* evaluation of [^123^I]**50** and its iodine-125 analog [^125^I]**52** [53] (Scheme 27).

*Reagents and conditions*: (**a**) [^123^I]NaI in 0.1% NaOH for [^123^I]**50** and [^125^I]NaI in 0.1% NaOH for [^125^I]**52**, CH_3_COOOH, MeOH, r.t., 5 min.

**Synthesis:** The synthesis of both radiotracers was performed in analogy to the earlier presented procedure [52] but in case of [^125^I]**52** purification was achieved by fractionized elution from SPE cartridges. This gave [^125^I]**52** in 90% decay corrected radiochemical yield and > 98% radiochemical purity within 30 min total synthesis time and [^123^I]**50** in the same yield and purity within 15 min total synthesis time. *In vitro*: Cell uptake studies were performed with [^125^I]**52** and it turned out that the cell uptake of [^125^I]**52** was increased in NCI-H322 human lung cancer cells in comparison to untreated cells when incubated at the same time with the nicotine-derived nitrosamine COX-2 inducer 4-(methylnitrosamino)-1-(3-pyridyl)-1-butanone (NNK). Pre-incubation with celecoxib significantly reduced the uptake in stimulated as well as unstimulated cells. *In vivo*: *In vivo* studies were performed with Syrian golden hamsters. Biodistribution studies with [^125^I]**52** in NNK treated and untreated animals revealed for all animals a time-dependent increase of tracer uptake in blood, lungs, liver and pancreas within 2 h. Interestingly, the uptake in NNK treated animals was 2.3 and 2.9 fold increased in lungs and liver and 11 fold increased in pancreatic tissue in comparison to the untreated animals. Whole body planar nuclear SPECT imaging with [^123^I]**50** in animals treated for 10 weeks with NNK revealed that radioactivity of [^123^I]**50** was accumulated in pancreas and liver when these tissues expressed COX-2 which was confirmed by immunohistochemical staining. In contrast, in untreated control animals [^123^I]**50** remained at the injection site. Post mortem, none of CO_2_-euthanized animals showed uptake of [^123^I]**50** in the lungs although extensive damage of lung tissue was shown. The authors suggested that this was caused by carbon dioxide-related pulmonary hyperacidosis what may have destroyed COX-2. In contrast, for one of two animals euthanized by anesthetic overdose uptake of radioactivity in the right lung was found. In this case, immunohistochemical staining revealed one COX-2 positive adenoma and eight COX-2 positive microadenomas in the right lung. [^123^I]**50** did not accumulate in the intestine of any hamster what revealed its specificity for COX-2 because there was found a pronounced base level of COX-1 expression.

#### 2009

**Scheme 28:**
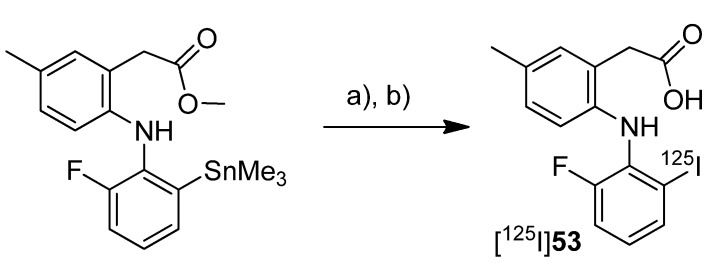
Synthesis of [^125^I]**53**.

Three years after the synthesis of [^125^I]**51** and [^125^I]**52**, Kuge *et al.* published the synthesis and evaluation of the radioiodinated lumiracoxib derivative 2-(2-((2-fluoro-6-[^125^I]iodophenyl)amino)-5-methylphenyl)acetic acid ([^125^I]**53**, Scheme 28) [80].

*Reagents and conditions*: (**a**) [^125^I]NaI in 0.2 M NaOH, H_2_O_2_, HCl/EtOH, r.t., 5 min; (b) NaOH, 40 °C, 15 min.

The COX-inhibition potency of **53** was determined by colorimetric assay as previously described and revealed an IC_50_ for COX-2 of 2.46 µM and for COX-1 of 446 µM. **Synthesis:** [^123^I]**53** was synthesized by radioiodination of the trimethylstannylated precursor and subsequent hydrolysis of the methyl ester. Briefly, no carrier added sodium [^123^I]iodide was stirred with the precursor in presence of H_2_O_2_ for 5 min at room temperature, the reaction was terminated by addition of NaHSO_3_ and after basifying with NaOH to pH = 9.0 alkaline saponification was performed for 15 min at 40 °C. Purification by HPLC gave [^123^I]**53** in 36–51% radiochemical yield (n = 3) with a specific activity of 47–72 GBq/µmol and radiochemical purity of > 95%. The log *D*_7.4_ was determined to be 1.84 ± 0.01 (n = 3) for [^123^I]**53** and 3.09 ± 0.11 (n = 3) and 2.97 ± 0.01 (n = 3) for [^123^I]**51** and [^123^I]**52**, respectively, which served as reference. ***In vitro*:** Cell uptake studies were performed with JA-4 cells subcloned from murine macrophage-like cell line J774.1 as control cell line with low basal COX-2 expression and LPS/IFN-γ-stimulated JA-4 cells with upregulated COX-2 expression as confirmed by Western blot analysis. [^123^I]**53 **uptake was significantly higher in LPS/IFN-γ-stimulated macrophages in comparison to the control and decreased by addition of the nonradioactive reference while uptake in the control was not affected. ***In vivo*/*Ex vivo*:** Male Sprague-Dawley rats were used for biodistribution studies. This revealed that [^123^I]**53** showed fast blood clearance, a high but with the time decreasing accumulation in the liver and the kidneys and a gradually increasing level in the intestine. The brain, stomach and thyroid did not show significant accumulation of radioactivity, the latter indicated the stability of [^123^I]**53** in case of de[^125^I]iodination. The accumulation in the kidneys was consistent with the *in vivo* expression of COX-2 and the low brain accumulation was consistent with the low lipophilicity of the radiotracer.

**Scheme 29:**
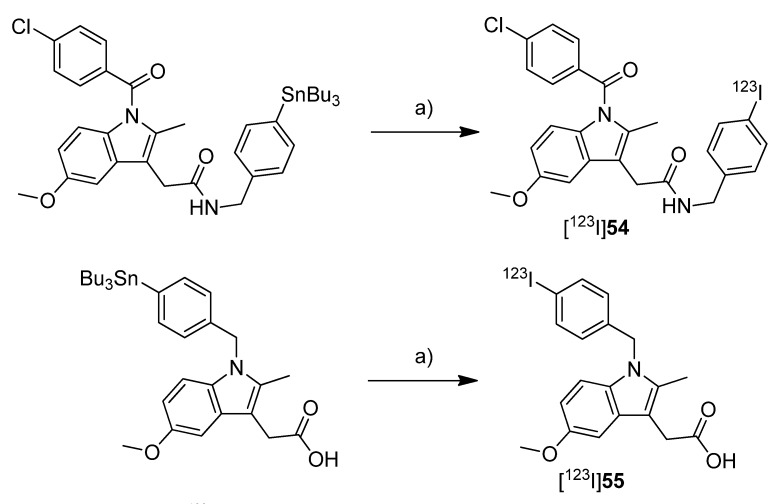
Synthesis of [^123^I]**54 **and [^123^I]**55**.

Uddin *et al.* published in 2009 the synthesis and *in vivo* evaluation of 2-(1-(4-chlorobenzoyl)-5-methoxy-2-methyl-1*H*-indol-3-yl)-*N*-(4-[^123^I]iodobenzyl)acetamide ([^123^I]54, Scheme 29) and 2-(1-(4-[^123^I]iodobenzyl)-5-methoxy-2-methyl-1*H*-indol-3-yl)acetic acid ([^123^I]55, Scheme 29), two iodine-123-labeled indomethacin derivatives [49]. Both compounds were selected from a set of nonradioactive compounds synthesized and evaluated in this work for their COX-inhibition potency. Non-radioactive references 54 and 55 showed potent and selective COX-inhibition with IC_50_ values of 0.12 µM and 0.40 µM, respectively, for recombinant human COX-2 and > 66 µM for purified sheep COX-1 as well as inhibition of COX-2 in intact RAW 264.7 cells with an IC_50_ value of 1 µM and 0.08 µM, respectively. Furthermore, both compounds were characterized as slow, tight-binding inhibitors. In addition, 55 was evaluated for its *in vivo* stability using female nude mice with 1483 human head and neck squamous cell carcinoma xenografts expressing COX-2. Compound 55 was injected retroorbitally and the analysis of tumor tissue revealed that 55 was found to be intact in the tumor (3 nmol/g tissue). **Synthesis:** [^123^I]54 and [^123^I]55 were synthesized from the corresponding tributylstannyl derivatives by iododestannylation in a binary phase system. For this, carrier added sodium [^123^I]iodide was reacted for 4 min at room temperature with the precursor in a mixture of ethyl acetate/water in the presence of chloramine-T (CAT) and hydrochloric acid. For purification, the organic phase was separated, washed with water and evaporated what yielded [^123^I]54 and [^123^I]55 in 86–87% decay corrected radiochemical yield with a specific activity of 18.2 GBq/µmol and radiochemical purity of 98–99%.

*Reagents and conditions*: (**a**) [^123^I]NaI in 0.01 M NaOH, NaI, CAT·3 H_2_O, 1 M HCl, H_2_O/EtOAc, r.t., 4 min.

#### 2010

**Scheme 30:**
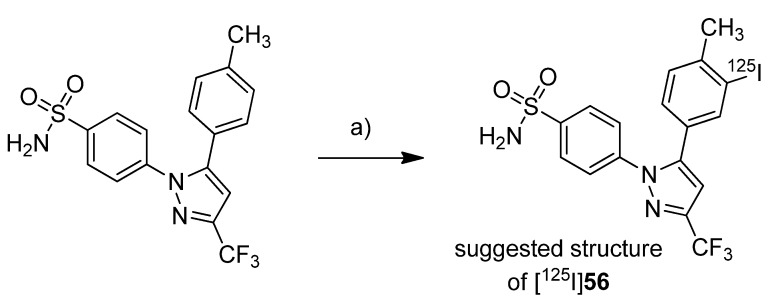
Synthesis of [^125^I]**56**.

In 2010, El-Azony presented the synthesis and biodistribution of a radioiodinated celecoxib derivative synthesized by reaction of celecoxib with sodium [^125^I]iodide and chloramine-T [81]. The non-radioactive reference was not described in this work so that the author points out that the product is presumably 4-(5-(3-([^125^I]iodo)-4-(methyl)phenyl)-3-(trifluoromethyl)-1*H*-pyrazol-1-yl)benzene-sulfonamide ([^125^I]**56**, Scheme 30).

*Reagents and conditions*: (**a**) [^125^I]NaI in NaOH (pH = 7–11), CAT, EtOH, 60 °C, 15 min.

**Synthesis:** The radiolabeling procedure started from celecoxib and experiments revealed as optimized conditions a celecoxib concentration of 3.9 mM, a chloramine-T concentration of 1.6 mM, that *N*-bromosuccinimide was not as effective as chloramine-T, a reaction time of 30 min, a pH = 4 and a temperature of 60 °C. Using the optimized conditions and a reaction time of 15 min, [^125^I]**56** was synthesized in about 65% labeling yield, purified by HPLC and formulated. ***In vitro*:** The tracer was stable for 24 h after formulation in saline solution. ***In vivo*:** For the *in vivo* evaluations were used Ehrlich ascites carcinoma bearing female Swiss albino mice which showed either ascites tumors or solid tumors. The biodistribution studies were performed 30 min, 1 h, and 24 h after injection of [^125^I]**56** into the tail vein what revealed increasing uptake over the time as well as highest uptake of 55% injected dose per gram of (ID/g) tissue after 24 h in the ascitic fluid. Furthermore, it was found a high radioactivity uptake in the kidney which gradually decreased presumably reflecting excretion via urine. A low amount of radioactivity was found in the thyroid and gradually decreasing amounts were found in other organs like blood, stomach and liver.

#### 2011

**Scheme 31:**
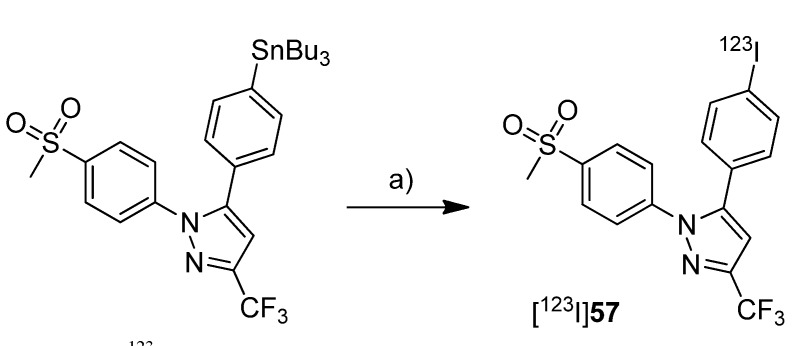
Synthesis of [^123^I]**57**.

Two years after the synthesis of [^123^I]**54** and [^123^I]**55**, Uddin *et al.* presented the synthesis of 5-(4-[^123^I]iodophenyl)-1-(4-(methylsulfonyl)phenyl)-3-(trifluoromethyl)-1*H*-pyrazole ([^123^I]**57**, Scheme 31), an iodine-123-labeled celecoxib derivative, and its evaluation with an inflammation model [50]. The synthesis and biodistribution of the analog iodine-125-labeled inhibitor [^125^I]**51** was described by Kuge *et al.* [39] (see above).

*Reagents and conditions:* (**a**) [^123^I]NaI in 0.01 M NaOH, NaI, CAT·3 H_2_O, 1 M HCl, H_2_O/EtOAc, r.t., 4 min.

[^123^I]**57** was selected due to the high and selective COX-inhibition potency of its nonradioactive reference possessing an IC_50_ value of 0.05 µM for purified murine COX-2 and > 4 µM for ovine COX-1 and an IC_50_ value of 0.03 µM for COX-2 in intact RAW 264.7 cells as well. **Synthesis:** [^123^I]**57** was synthesized by iododestannylation analogously to the synthesis protocol for [^123^I]**54** and [^123^I]**55** with minor modifications. This gave [^123^I]**57** in 85% radiochemical yield and 99% radiochemical purity with a specific activity of 18.2 GBq/µmol. ***In vivo*:** For the *in vivo* evaluation of [^123^I]**57**, inflammation was induced by injection of carrageenan into the rear hind paw of Sprague-Dawley rats and 3 h later [^123^I]**57** was administrated by injection into the tail vein. Combined small animal SPECT/CT was performed 2 h after injection and revealed that radioactivity from [^123^I]**57** was selectively taken up in the swollen paw. Furthermore, biodistribution studies showed that the uptake of radiotracer at 3 h post-injection in the swollen paw was 1.5 times higher than in the control paw. This uptake could be decreased to 1.0 ± 0.2 by blocking with pre-administrated nonradioactive reference. In summary, the uptake of [^123^I]**57** in the inflamed and non-inflamed paw of the animal was determined to be 23.5 ± 5% and 11.5 ± 5% ID/g organ, respectively.

#### 2012

**Scheme 32:**
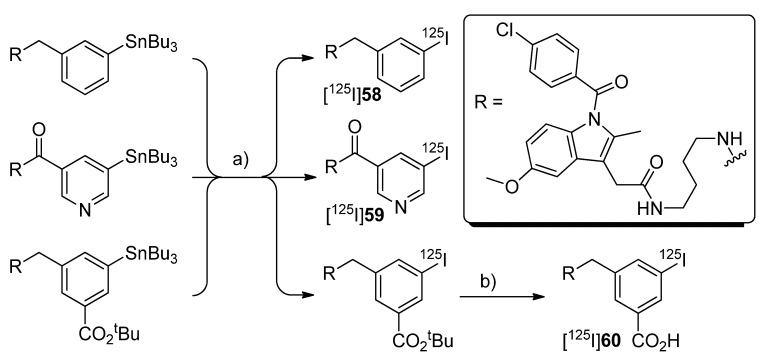
Synthesis of [^125^I]**58**–**60**.

Very recently, Zlatopolskiy *et al.* presented in a conference contribution the synthesis and *in vitro* evaluation of the indomethacin amides [^125^I]**58**–**60** [82] (Scheme 32).

*Reagents and conditions*: (**a**) [^125^I]NaI, CAT; (**b**) deprotection.

**Synthesis:** [^125^I]**58**–**60** were synthesized by radioiodination starting from the corresponding SnBu_3_-substituted precursors with chloramine T as an oxidant and in case of [^125^I]**58** by subsequent deprotection in 46-80% radiochemical yield. For further evaluation [^125^I]**58**–**60** were formulated in 50% ethanol. ***In vitro*:** Cell uptake studies were performed which revealed that the uptake of 2-(1-(4-chlorobenzoyl)-5-methoxy-2-methyl-1*H*-indol-3-yl)-*N*-(4-((3-[^125^I]iodobenzyl)amino)butyl)acetamide ([^125^I]**58**) and *N*-(4-(2-(1-(4-chlorobenzoyl)-5-methoxy-2-methyl-1*H*-indol-3-yl)acetamido)butyl)-5-[^125^I]iodonicotinamide ([^125^I]**59**) was increased in HEK hCOX-2 cells with induced COX-2 expression and that the uptake of [^125^I]**58** was increased in TPA- stimulated HUVEC cells. This correlated with the COX-2 expression pattern determined by Western blot analysis. In contrast, 3-(((4-(2-(1-(4-chloro-benzoyl)-5-methoxy-2-methyl-1*H*-indol-3-yl)acetamido)butyl)amino)methyl)-5-[^125^I]iodobenzoic acid ([^125^I]**60**) showed only low uptake in COX-2 expressing HEK cells. 

**Figure 2:**
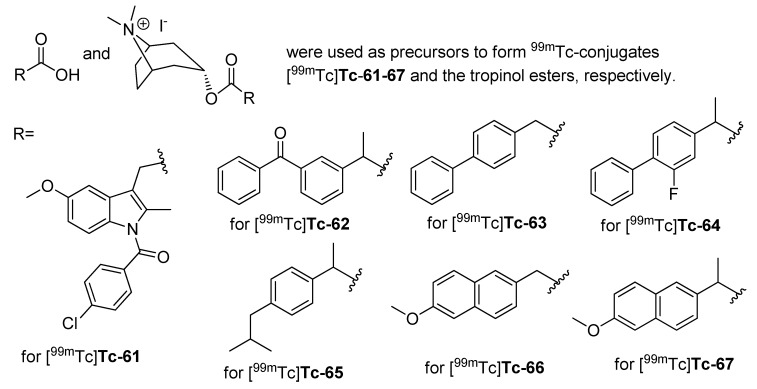
Structures of the labeling precursors for the synthesis of [^99m^Tc]**Tc-61**–**67**.

Finally, technetium-99m-labeled COX inhibitors have been synthesized, too. In 2008, technetium-99m-labeled conjugates of indomethacin ([^99m^Tc]**Tc-61**), ketoprofen ([^99m^Tc]**Tc-62**), biphenylacetic acid ([^99m^Tc]**Tc-63**), flurbiprofen ([^99m^Tc]**Tc-64**), ibuprofen ([^99m^Tc]**Tc-65**), 6-methoxy-2-naphtylacetic acid ([^99m^Tc]**Tc-66**), naproxen ([^99m^Tc]**Tc-67**), and the corresponding technetium-99m-labeled tropinol-NSAID esters were presented by Yadav *et al.* [83,84]. The chemical structure of the complexes finally formed was not presented by the authors. Instead, the chemical structures of the precursors were given which are depicted in Figure 2 for a better overview. **Synthesis:** The complex formation was performed with sodium [^99m^Tc]pertechnetate and stannous chloride at pH = 7 for 30 min what yielded the conjugates in 88-98% radiochemical yield. ***In vivo*:** Small animal SPECT imaging was performed in Sprague-Dawley rats after induction of inflammation by subcutaneous injection of carrageenan. All tested technetium-99m-labeled NSAIDs were not able to detect the inflamed joint tissue, in contrast to the technetium-99m-labeled tropinol ester conjugates due to the affinity of quaternary amines to the cartilaginous tissue. 

**Figure 3:**
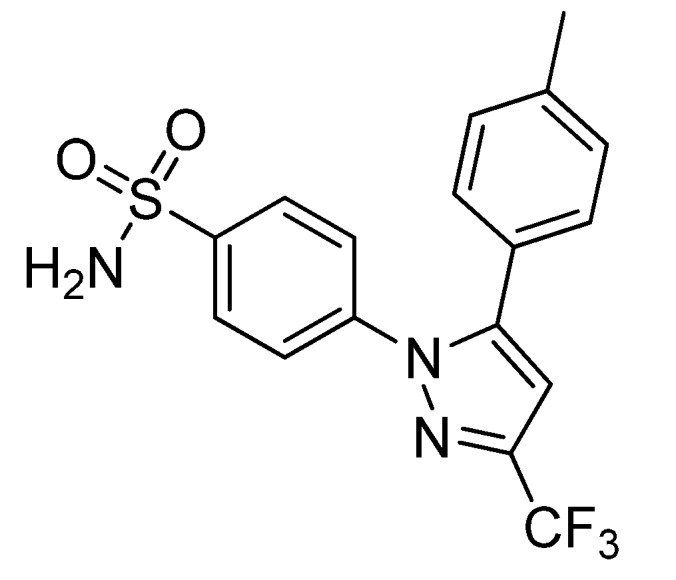
Structure of celecoxib, the labeling precursor for the synthesis of [^99m^Tc]**Tc-68**.

In 2011, Farouk *et al.* presented the results of the radiosynthesis and biodistribution study of technetium-99m-labeled celecoxib ([^99m^Tc]**Tc**-**68**) [85]. The chemical structure of the complex finally formed was not presented by the authors. For a better overview, the chemical structure of celecoxib is depicted in Figure 3. **Synthesis:** The labeling procedure with sodium [^99m^Tc]pertechnetate was optimized what revealed best yields under the following conditions: 500 µg of stannous chloride dihydrate, 500 µg of celecoxib, the pH between 5 and 9, a reaction time of 30 min, and room temperature. In this protocol, celecoxib was labeled at pH = 7 in 99.7% radiochemical yield. The synthesized complex was stable for 24 h after formulation in saline solution. ***In vivo*/*Ex vivo*:** Biodistribution studies in Albino Swiss mice revealed that the level of [^99m^Tc]**Tc**-**68** in blood and liver gradually decreased within 4 h post injection and a high uptake in the kidneys indicating clearing from systematic circulation and excretion to the urine. Biodistribution was furthermore studied in mice after induction of sterile inflammation with sterile turpentine in the muscle. [^99m^Tc]**Tc**-**68** uptake in the inflamed muscle gradually increased in comparison to the non-inflamed muscle and was 2.4 fold higher in the inflamed muscle after 4 h. The authors suggested that this was due to the high vascularization in the inflamed area and, thus, the better blood supply at this side. 

## 3. Discussion

Within the last decade, noticeable efforts were undertaken to develop compounds that are able to visualize functional expression of COX-2 *in vivo* by means of PET or SPECT. The result is a library of radiolabeled compounds. Remarkably, more than 60 novel radiolabeled COX inhibitors distinguishable from each other by chemical structure or radionuclide were developed since 2005 and most of them derived from leads that selectively inhibit COX-2 with high affinity *in vitro* or even *in vivo*. Although not all tracers have been fully investigated *in vitro* and/ortested *in vivo*, and only some showed promising results, we have learned a lot about criteria influencing the potential of radiotracers for imaging of COX-2 expression. 

Generally, the *selectivity and affinity for COX-2* is considered as the initial criterion for the selection of potential leads to visualize COX-2 expression [43]—that means a lead for a potential radiotracer should demonstrate an affinity for COX-2 in the nanomolar range together with a high selectivity. Fortunately, a variety of COX-2 inhibitors are described in the literature. However, the majority of radiotracers were derived from potent drugs that are in the clinical use. Of note, some of them have been already withdrawn from the market due to substantial adverse side effects when applied in long-term treatment. In detail, a set of compounds are derivatives of the clinically available NSAID indomethacin ([^11^C]**39**–**43**, [^18^F]**47**–**49**, [^123^I]**54**–**55**, and [^125^I]**58**–**60**), and of the COXIB celecoxib ([^11^C]**7**, [^11^C]**9**, [^18^F]**16**, [^18^F]**30**, [^11^C]**32**–**38**, [^123^I]**50**–**52**, [^125^I]**56**, [^123^I]**57** and [^99m^Tc]**Tc-68**). Furthermore, derivatives of the well-known COXIBs eterocoxib ([^11^C]**5**), lumiracoxib ([^125^I]**53**), valdecoxib ([^18^F]**3**), and rofecoxib ([^18^F]**2**, [^11^C]**6**) have been radiolabeled. Indeed, these COXIBs are highly selective inhibitors with high affinity for COX-2 that have been extensively studied in clinical trials and should therefore represent suitable leads in the first place. Due to the fact that the applied dose of a radiolabeled drug for a radiotracer examination is in the magnitude of a few nanomol or lower, its selectivity and affinity are of highest importance and other pharmacological properties like toxicity and dose-response relationship are fading into the background. Indeed, compounds which have already failed in clinical studies due to toxicity or adverse side effects as well as compounds that have not yet been clinically evaluated as a drug may also represent potent candidates for radiolabeling. As examples may be listed compounds like diaryl substituted cyclopentene ([^18^F]**1**, [^11^C]**17**), pyran-2-one ([^18^F]**12**), imidazole ([^11^C]**13**–**14**) and indole ([^11^C]**15**, [^18^F]**45**–**46**) derivatives which have not been evaluated in clinical studies but which have been tested as radiolabeled COX-2 inhibitors. The IC_50_ value for COX-1 and COX-2 was used as criterion for the affinity and isoform selectivity of COX-inhibitors and is expected for tracers targeting COX-2 to be advantageous in the low nanomolar range. It should be noted that the determined value of the IC_50_ strongly depends from the experimental protocol and the type of assay used. For example, for 5-(4-iodophenyl)-1-(4-(methylsulfonyl)phenyl)-3-(trifluoromethyl)-1*H*-pyrazole (**51**/**57**) an IC_50_ for ovine COX-2 of 5.16 µM was determined by using a colorimetric assay [39]. If the same compound was evaluated with a [^14^C]arachidonic acid based assay system it gave an IC_50_ for human COX-2 of 0.05 µM [50]. Another example is 3-(4-fluorophenyl)-2-(4-(methylsulfonyl)phenyl)-1*H*-indole (**45**) which showed in a radioimmunoassay based cell assay an IC_50_ for COX-2 in activated macrophage cells of 0.02 nM [66]. In contrast, if the same compound was evaluated with a fluorescence based assay it revealed an IC_50_ for recombinant human COX-2 of only 1.2 µM [31]. For that reason, it is essential to compare the IC_50_ values of the test compounds with a standard like celecoxib or indomethacin within the same assay to avoid misinterpretation. Additionally, it seems to be advantageous to study the *enzyme-inhibitor binding kinetics* because the binding of the tracer [^18^F]**30** at COX-2 expressing sites was related to its kinetic properties as slow, tight binding inhibitor [48]. Beside this, the radiosynthesis of unselective or COX-1 selective inhibitors ([^11^C]**18**–**29**, [^11^C]**44**, [^99m^Tc]**Tc-61**–**67**) was also presented in the literature. However, although the selectivity and affinity are prominent criteria these are not the only determinants for a suitable radiotracer for COX-2 since, e.g., compounds **9** [51], **17** [30], and **41** [35] also exhibit an IC_50_ for COX-2 in the nanomolar range but the corresponding radiotracers did not show specific binding *in vivo*. 

The *radiochemical* and *in vivo* *stability* of the radiolabeled compound is another eminently important characteristic of a radiotracer [48]. The tracer should be stable at least for the time interval of the radiopharmacological examination and therefore the *in vitro* and/or *in vivo* stability was examined at least for the most tracers. The results showed *in vivo* de[^18^F]fluorination for tracers with [^18^F]fluoro-alkyl bonds ([^18^F]**3** [33], [^18^F]**16** [44]) as well as fast metabolism for tracers with [^11^C]methoxy-functionality ([^11^C]**15** [43], [^11^C]**17** [30])or compounds having an ester ([^11^C]**22 ** used as prodrug [45], [^11^C]**39** [35]) or amide ([^11^C]**40**–**43** [35]) functionality. The [^11^C]methylphenyl substituted compound [^11^C]**7** was found to be metabolized to its carboxylic acid metabolite [^11^C]**32** in the bile [75]. In contrast, [^18^F]**45** with a [^18^F]fluorine-phenyl bond showed no de[^18^F]fluorination and acceptable *in vivo* stability. The iodine-phenyl bond of the radioiodine labeled compounds ([^125^I]**51**-**52** [39], [^123^I]**53** [80]) proved to be stable at least for deradioiodination as indicated by a lack of accumulation of radioiodine in the thyroid. For [^18^F]**30** the potential for defluorination was examined by LC-ESI-MS with the non-radioactive reference. This kind of metabolite analysis revealed 2 h post injection an extent of 8.8 ± 5% (n = 3) of a defluorinated metabolite in the tissue of interest [48]. Similarly, tumor uptake of intact **55** was shown by HPLC analysis 3 hours post retroorbital injection [49]. However, it is questionable whether the *in vivo* stability of the non-radioactive probe can serve as reference for the stability of the radiolabeled compound under the same conditions because of the high concentration difference. Furthermore, the sensitivity of the radiotracer toward radiolysis should be examined and, if this is the case, it should be prevented by appropriate synthetic strategies, e.g., radiosynthesis with lower starting activities or addition of antioxidants as shown for compounds [^11^C]**18**, [^11^C]**19**, and [^11^C]**22**–**24** [45,72]. The *lipophilicity* of the tracer and its interaction with target tissues should be considered as important criterion for COX-2 imaging agents, too. In general, the COX-2 active site and the lipophilic interactions of the inhibitors with the enzyme presuppose a certain lipophilicity of the tracer [35]. For crossing the blood brain barrier as well as cell membranes [31,48], a certain lipophilicity is a prerequisite. However, with increasing lipophilicity the tendency for binding to serum albumin or other plasma proteins [35] and, additionally, the so called non-specific binding in adipose tissue and deposits [30] increases as well. For example, an inverse correlation between increasing lipophilicity and brain uptake was found for compounds [^11^C]**39**–**43** presumably due to plasma binding effects [35]. 

A lot of COX-2 selective inhibitors share the diaryl heterocyclic core structure displaying a common structural motif in this inhibitor class. For these compounds, the feasibility to inhibit COX-2 selectively depends on the appearance of an *aminosulfonyl* (**SO_2_NH_2_**) or a *methylsulfonyl* (**SO_2_CH_3_**) group. This moiety was identified as important determining factor for the specificity of the radiotracer. By a direct comparison it was demonstrated that the methyl sulfonyl substituted celecoxib derivative [^125^I]**51** showed a faster blood clearance than its amino sulfonyl substituted analog [^125^I]**52**, an effect that was related *in vitro* to the affinity of the enzyme carbonic anhydrase to the amino sulfonyl function [39]. To what extent the *half-life* of the used radionuclide is a determinant for a successful COX-2 radiotracer accumulation has been discussed [39,86] but has not been explicitly determined yet. In general, the very short half-life of carbon-11 (20 min) is a limiting factor for an elaborated PET investigation. In contrast, fluorine-18 (110 min), besides its outstanding properties as PET nuclide, provides a more suitable time window for both radiosynthesis and subsequent dynamic PET studies also regarding physiological factors like tissue vascularization and perfusion. However, in certain physiological situations, radionuclides exhibiting longer physical half-life will be needed. Here, radioisotopes of iodine would be the better choice. Of note, up to now no iodine-124 radiolabeled selective COX-2 inhibitor suitable for PET has been developed.

Finally, the *specific activity* of the radiolabeled compounds determines the feasibility as radiotracer. As a rule, a high specific activity is requested to minimize binding competition between the labeled and the non-radioactive inhibitor [31]. For carbon-11-labeled compounds specific activities between 14 ± 8 GBq/µmol for [^11^C]**6** [29] and 277.5 ± 92.5 GBq/µmol for [^11^C]**9 ** and [^11^C]**33**–**38** [46] have been reported. In comparison, for fluorine-18-labeled compounds specific activities were observed in the range of 4.44 ± 1.48 GBq/µmol for [^18^F]**16** [44] and 74–91 GBq/µmol for [^18^F]**45** [31], and were reported to be 18.2 GBq/µmol for [^123^I]**57** [50] and 47–72 GBq/µmol for [^125^I]**53** [80] in case of radioiodine labeled COX inhibitors. 

Another important criterion for the evaluation of radiolabeled COX-2 inhibitors is the choice of the most suitable system for the *in vitro* and especially the *in vivo* evaluation. Up to now, there is no appropriate standard model available. Several *cell models* have been used for the *in vitro* evaluation of radiolabeled COX-2 inhibitors but the results did not unambiguously correlate with the *in vivo* behavior of the compounds. For example, [^11^C]**17** [30] and [^18^F]**45** [31] showed specific binding to a set of cell lines with low or increased COX-2 expression *in vitro*; among them, COX-2 expressing HT-29 human colorectal adenocarcinoma cells. However, this specificity could not be confirmed *in vivo* for COX-2 expressing HT-29 tumor xenograft bearing nude mice. In contrast, for [^125^I]**52** an increased uptake in NNK-induced COX-2 expressing cells as well as a partially reduced uptake by blocking with celecoxib was demonstrated that was consistent with an increased uptake of [^125^I]**52** observed in COX-2 overexpressing tissues of NNK-treated animals [53]. 

The *in vivo* *biodistribution* of radiolabeled COX-2 inhibitors in *animals showing physiological expression of COX-2* was examined by organ excision, PET or SPECT imaging, respectively, with the aim to characterize the tracer distribution as well as to find retention in tissues with constitutive COX-2 expression like kidney, heart, intestine, and brain. By that, a hepatobiliary elimination of the tracers as indicated by high uptake in gall bladder/bile, liver and intestine was found for [^18^F]**3** [33], [^18^F]**45** [31], [^11^C]**39**–**43** [35], [^11^C]**7** [75], [^11^C]**17** [30] and can be supposed for [^125^I]**51**–**53**, too. Furthermore, non-specific binding to adipose tissue was observed for [^11^C]**15** [30] in rats and for [^18^F]**45** [31] in mice. A high bone uptake of [^18^F]**3** in mice and baboon indicated de[^18^F]fluorination of the radiotracers applied [33]. An non-specific uptake in the brain of mice or rats was observed for [^11^C]**39**–**40** [35], [^11^C]**42**–**43** [35], [^11^C]**7** [67] and [^11^C]**17** [35], the latter showed high initial uptake but little retention, and revealed to be very low for [^125^I]**53** [80]. Yet, it was not shown whether the heterogeneous uptake of [^18^F]**3** into the brain of baboon [33] and the uptake of [^125^I]**51**–**52 **into the brain of rats [39] was specific. In contrast, [^11^C]**6** showed specific distribution in the brain of rats [29], and [^11^C]**41** gave a small fraction of specific brain uptake in mice [35]. Higher levels of radiotracers in blood ([^11^C]**7** [67], [^11^C]**33** [46]) or single organs like lung ([^11^C]**7** [67], [^125^I]**51**–**53** [39,80], [^11^C]**33** [46]), kidneys ([^125^I]**53** [80], [^11^C]**33** [46]), heart ([^125^I]**51**–**52** [39]) or the harderian glands ([^18^F]**45** [31]) have been also pointed out but the specificity was not examined. Due to these heterogeneous results, the possibility to show specific distribution of COX-2 tracers to tissues with physiological COX-2 expression and the performance of corresponding blocking studies was discussed [39,80].

Finally, radiolabeled COX-2 inhibitors have been evaluated *in vivo* by different *animal models showing overexpression of COX-2*. In PET studies, there was observed no uptake of [^18^F]**45** in the HT-29 tumor xenograft and a visible uptake of [^11^C]**17** in the HT-29 tumor xenograft that could not be blocked [30,31]. Biodistribution studies of [^11^C]**6** revealed enhanced but not significant brain uptake in herpes simplex virus infected rats as well as no increased radiotracer uptake to inflammatory sites in animals with turpentine induced sterile inflammation [29]. For AH109A hepatoma tumor xenograft bearing mice, biodistribution studies revealed an accumulation of [^11^C]**8**–**9** and [^11^C]**13**–**15** in the liver and the small intestine indicating an hepatobiliary excretion, together with a low brain uptake [43,51]. Blocking studies performed with carrier loading, celecoxib or NS-398 showed significant blocking effects for [^11^C]**8** with carrier loading in lung, spleen, and small intestine and with indomethacin in liver, spleen, and small intestine, as well as for [^11^C]**9** with carrier loading in spleen and blood. In contrast, no significant blocking effect was found in case of [^11^C]**13** [^11^C]**14** and [^11^C]**15**. In the tumor tissue [^11^C]**8**–**9 **as well as [^11^C]**13**–**15** showed only low and non-specific uptake. That means for HT-29 tumor xenograft bearing mice, AH109A tumor xenograft bearing rats, Herpes simplex virus infected rats, and rats with turpentine induced sterile inflammation a specific uptake could not be demonstrated with the tested tracers. In this regard, the influence of *efflux transporters* like P-glycoprotein (P-gp) was examined what revealed that [^11^C]**7** was no substrate of P-gp [67] and evaluations e.g., for, [^11^C]**15** [43], [^11^C]**39**–**43** [35], [^18^F]**45** [31] did not give a clear result. By whole body planar SPECT imaging of hamsters pretreated with the COX-2 inducer NNK, an increased uptake of [^123^I]50 and [^125^I]52 was demonstrated at organs with COX-2 overexpression [53]. In comparison, no uptake was found in organs that do not express COX-2 and in untreated animals, respectively. In this regard, also the low dose of 3.7 MBq used for one study was discussed as criterion allowing the selective differentiation of organs with significantly overexpressed COX-2 levels from those with physiologically slightly elevated levels [53]; however, the specificity of this approach was not proven by blocking studies yet. 

Fortunately, also successful examples have been reported for COX-2 specific radiotracer development that means an uptake in COX-2 overexpressing tissues connected with the successful *proof of specificity by blocking in vivo*. By using an carrageenan-induced inflammation model, [^123^I]**57** showed an 1.5 fold uptake in the inflamed paw in comparison to the non-inflamed paw that could be blocked by pre-administration with the nonradioactive reference [50]. Also, using the ^18^-radiolabeled COX-2 inhibitor [^18^F]**30** on a carrageenan-induced inflammation model as well as on a 1483 HNSCC tumor model an uptake saturable by pre-administration with celecoxib, and hence specific uptake, could be demonstrated by the same authors [48]. In detail, [^18^F]**30** showed enhanced specific uptake in inflammatory sites induced by carrageenan in wild type Wistar rats and wild-type mice but not in COX-2 null mice and consistently, a specific uptake in COX-2 expressing 1483 HNSCC tumor xenografts but not in COX-2 negative HCT116 tumor xenografts [48]. 

Hence, though targeting of COX-2 is complicated by the histological localization of the enzyme in the endoplasmatic reticulum of the cell [43] as well as the fact that absolute expression of the enzyme in physiological and pathophysiological situations is uncertain [29], nowadays *in vivo/in vitro* models for the characterization of radiolabeled COX-2 inhibitors are available. Because up to now some tracers have not been evaluated *in vivo/in vitro* or have failed to show specific binding *in vivo* although *in vitro* results have been promising, it would be now a firm goal to develop an *in vivo/in vitro* standard model for the comparison of the tracers. This would help to explain to what extent some synthesized radiolabeled COX-2 inhibitors failed; due to the shortcomings of the tracers themselves or due to the limitations of the *in vitro* or *in vivo* models used [29,80].

## 4. Conclusions

Non-invasive radionuclide-based imaging techniques like PET and SPECT have the potential to substantially improve the diagnosis and therapy of several diseases given that the suitable biological target and the corresponding radiotracer is available. In this regard, the inducible cyclooxygenase isoform COX-2 is discussed as promising target for the diagnosis as well as for the treatment of inflammation-associated processes and cancer. This is due to its favorable expression pattern, its important role in the development and progression of inflammatory and neoplastic disorders and the possibility to address this enzyme selectively by specific inhibitors with high affinity. 

For this, radiolabeled selective COX-2 inhibitors are discussed to be very promising probes. Within the last decade more than 60 compounds have been radiolabeled with the PET isotopes fluorine-18 and carbon-11 and with radioiodine as well. Although a majority of radiolabeled COX-2 inhibitors were not yet investigated biologically or after promising *in vitro* evaluation failed to demonstrate specific binding *in vivo*, we have learned some lessons about this class of radiotracers and cyclooxygenases as molecular target. High affinity down to the nanomolar level and high selectivity to the enzyme are only some aspects for tracer development, factors like lipophilicity and metabolic stability also have to be considered. Many of the tested compounds did not show sufficient stability *in vivo* due to de[^18^F]fluorination or de[^11^C]methylation or failed to bind specifically in the target region. The vascularization and the resulting perfusion of the tumor as well as edema formation in inflammatory sites were discussed to influence or even to counteract the tracer uptake in the target tissue. An unitary model for examination of COX-2 expression with radiotracers *in vitro*/ *in vivo* would help in the evaluation of future compounds and to gain information about their future potential or their failure, respectively.

Fortunately, some recent studies have finally demonstrated a specific uptake of radiolabeled COX-2 inhibitors in inflammatory lesions as well as in tumor xenografts and gave the proof of principle of successfully targeting COX-2 *in vivo* with radiotracers. This gives hope for further developments in this field which now should focus on the transfer of potential candidates into clinical practice. 
